# A general framework to link theory and empirics in opinion formation models

**DOI:** 10.1038/s41598-022-09468-3

**Published:** 2022-04-01

**Authors:** Ivan V. Kozitsin

**Affiliations:** 1 Laboratory of Active Systems, V. A. Trapeznikov Institute of Control Sciences of Russian Academy of Sciences, 65 Profsoyuznaya street, Moscow, Russian Federation 117997; 2 Department of Higher Mathematics, Moscow Institute of Physics and Technology, Institutsky lane 9, Dolgoprudny, Moscow Region, Russian Federation 141700

**Keywords:** Applied mathematics, Complex networks, Statistical physics

## Abstract

We introduce a minimal opinion formation model that is quite flexible and can reproduce a wide variety of the existing micro-influence assumptions and models. The model can be easily calibrated on real data, upon which it imposes only a few requirements. From this perspective, our model can be considered as a bridge, connecting theoretical studies on opinion formation models and empirical research on social dynamics. We investigate the model analytically by using mean-field approximation and numerically via Monte Carlo simulations. Our analysis is exemplified by recently reported empirical data drawn from an online social network. We demonstrate that the model calibrated on these data may reproduce fragmented and polarizing social systems. Furthermore, we manage to generate an artificial society that features properties quantitatively and qualitatively similar to those observed empirically at the macro scale. This ability became possible after we had advanced the model with two important communication features: selectivity and personalization algorithms.

## Introduction

Models of opinion dynamics (aka social-influence models) concern how individuals change their opinions as a response to information their receive from social environments. Understanding the processes of opinion dynamics is important due to its applications in many fields, including policy-making, business, and marketing. This branch of modeling is naturally interdisciplinary, attracting scholars from different fields, such as social psychology, control theory, and physics. Despite the theoretical side of these models having been seriously advanced, the problem of its applications to describing real social processes remains an important question^1–5^. This issue is rooted in the complex nature of the social systems. To be more precise, it is extremely difficult to calibrate the parameters of the underlying social-influence models, which operate with hardly formalizable entities. One prominent example is that of opinions themselves, which are intrinsic components of such models^5^.

The proliferation of online social networks (OSNs) has made it possible to identify the dynamics of users’ opinions on a large scale by applying machine learning techniques^6,7^. Combining these methods with tools elaborated in the field of social network analysis, one can obtain both the dynamics of opinions and information on social connections between individuals^8^. Further, recent research has proposed a methodology to identify not only the structure of ties but also their weights, which describe how well these ties conduct social influence (aka influence networks)^9^. This information may be effectively integrated into the existing social-influence models, enabling them to be calibrated, validated, and, further, make necessary predictions.

However, the step involving the integration of information gathered from OSNs into the models is hampered because there is substantial scope of the opinion dynamics models, and each model may require a specific data format. Hence, empirical data gathered through an experiment (most likely an expensive and time-consuming one) may be useful in one case but, unfortunately, unacceptable or requiring a lot of additional work with the data in other situations. Thus, each new dataset on opinion dynamics will potentially have a limited area of application in the sense that it could be investigated only by a restricted number of opinion formation models.

Therefore, we propose a quite general and, nonetheless, minimal model of opinion formation. On the one hand, the model is extremely flexible and can approximate a wide variety of the existing micro-influence assumptions and models. On the other hand, our model can be easily calibrated on empirical data, upon which it imposes a relatively small number of requirements. From this perspective, this model can serve as a bridge, connecting theoretical studies on opinion formation models on the one hand and empirical research on the other. We investigate the model analytically and numerically and exemplify our analysis with recently reported empirical data drawn from an OSN.

## Literature

Social-influence models are numerous, and it is extremely difficult to classify all of them correctly. However, most of them can be grouped into main classes to some extent. Within this paper, we concentrate on the micro-level (agent-based) models, whereby the opinions of every individual are initialized and can both change and cause changes^10,11^. In such models, the modeler analyzes how influence processes at the micro scale affect the resulting state of the social system at the macro scale. In contrast, so-called macro-level models describe the behavior of macroscopic variables, such as populations of individuals espousing particular opinions^12^.

The literature emphasizes three main classification criteria. (1) Time in the model: continuous^13^ or discrete^14^; (2) opinions: continuous^15^ or discrete^16,17^; and (3) micro assumption of social influence (see below). Because the empirical data are typically gathered in discrete time, we will focus hereafter on discrete-time models. Besides, we will assume that opinions are represented by scalar quantities describing individuals’ positions on a single issue. More complex situations could arise when several topics (logically connected or independent) are analyzed at once^18,19^. However, gathering data on individuals’ opinions on two or more topics simultaneously is a challenging and costly task. On this basis, we will focus on scalar opinions.

There are three main micro assumptions regarding social influence, built upon a continuous opinion space^2^. In that space, in the case of one-dimensional opinions, one can say that an individual’s opinion is affected by positive (aka assimilative) influence from a different opinion if the former moves towards the source of influence^20^. However, the literature on social psychology stipulates that if opinions are too distant, then the positive influence may not be accepted. Hence, the concept of bounded confidence has been introduced whereby only individuals espousing sufficiently close opinions may communicate^21,22^. In turn, if individuals’ opinions become more distant following communication, then such a mechanism is termed negative (aka dissimilative) influence^23,24^. Note that in the case of a discrete opinion space, these assumptions are rather meaningless unless opinion values are ordered.

The positive influence mechanism explains elegantly how individuals reach agreement, and bounded confidence can model a situation when a social system is characterized by persistent disagreement (opinion fragmentation), whereas negative influence is one of the possible mechanisms explaining opinion polarization—the process in which individuals’ opinions are stretched to the polar positions of the opinion space^25^. Thus, two camps of diametrically opposite opinions appear, a state of the social system that may have potentially dangerous consequences because it prevents democracy processes in general and consensus reaching in particular^26^. An important challenge in the field of opinion dynamics models is to determine the settings under which the model would be able to generate stable opinion polarization. Possible solutions here, apart from negative influence, are mass media^26^, social feedback processes^25^, social influence structure^27^, arguments exchange^28,29^, social identity^30^, external events^31^, or mistrust^32^. Recently, the issue of opinion polarization was successfully explained by using concepts from machine learning theory^33^.

In contrast to continuous-opinion models in which individuals’ opinions reflect attitudes towards a specific issue or person (say, political candidates or some controversial topics such as global warming) and thus may be measured on a continuous scale, discrete-opinion models investigate situations when people have to make a decision between a limited number of alternatives (for example, between two or more competing political parties, vaccines, languages, etc.)^16,17,34–39^. These alternatives may be introduced on an equal basis (such as those undertaken in the Voter or Axelrod models) but could also be arranged in some way. For example, in Ref.^40^, the authors introduced a triple opinion space in which supporters of two alternatives (leftists and rightists) occupy polar positions and cannot interact but are allowed to interact with centrists. In such models, interacting agents tend to reduce mutual opinion discrepancies by adopting the opinions of each other. Possibly the most simple opinion dynamics protocol can be found in the Voter model whereby in each iteration, a randomly chosen agent adopts the opinion of one of its neighbors^16^. However, some modifications of the Voter model allow for an agent to change their opinion after interacting with those who hold the same position—a phenomenon referred to in Ref.^38^ as anticonformity (in principle, anticonformity may be understood as a special case of negative influence). Besides, the noisy Voter model assumes that agents, regardless of their surroundings, may spontaneously change their views^34,39^*.* More complex opinion dynamics protocols can be found in the Majority rule^41^, Sznajd^17^, or q-Voter^38,42^ models.

Typically, opinion dynamics models consider how opinion evolves on quenched social networks. However, real social networks are not static but do change during our lives, adapting to our interests and social contexts. Despite the many factors that govern these network dynamics, including structural ones^43^ (e.g., number of friends—more popular people tend to be more attractive to others—, and number of common peers—the more friends two individuals share, the more probability that they will make a connection in the future) or demographical (because shared social contexts, such as a common city, school, or workplace, increase the chance of friendship), there is a well-documented phenomenon that individuals with similar opinions have an increased likelihood of becoming friends, other things being equal (aka selectivity)^44,45^. As such, social network evolution may significantly affect the structure of social influence processes and, thus, should not be neglected. To address this issue, a line of research devoted to modeling coevolutionary processes whereby social influence mechanisms are combined with the dynamics of social graphs appeared^46–49^.

The advent of OSNs has changed how information circulates around the world. As for now, people can obtain, without the many limitations and costs typical for older forms of communication, information that they want from the Web. Content that users receive from online platforms is conveyed and, importantly, often produced by the users themselves. Further, individuals can create their own information environment by selecting which account to follow. As a result, OSNs constitute an enabling scene for an unfolding of social influence processes, fostering our natural communication needs. However, these online ecosystems represent a biased environment—recent results suggest that initial seeds of followees affect what information neutral unbiased Twitter users will receive in the future (importantly, this outcome is not caused by the bias of the platform itself)^50^. It is worth noting that OSNs not only provide a space for communication but also affect with whom and how we communicate. The reason for this ability is rooted in filtering algorithms (aka personalization systems)^51^. These algorithms arrange the information space around users and suggest to them that content (and those potential friends) that will be most important (to us) from the algorithms’ point of view, of course^52,53^. Officially, these systems attempt to make the time users spend more efficient and profitable. Besides, due to cognitive constraints, individuals may not be able to handle all the information they receive in OSNs. Personalization systems pretend to be a solution here because they sort out the information environment of a user and decide which information should be given more priority according to the history of a user’s actions in OSNs. However, there are many concerns that such algorithms pursue the business purposes of OSNs’ holders; thus, their goal could be to maximize users’ time in OSNs. To achieve this goal, they may expose users to the content that will be guaranteed to be enjoyed. One way to achieve this aim is to isolate individuals from uncomfortable information and potential sources of such information. As a result, people are caught in so-called filter bubbles whereby they have no access to challenging content. This issue may prevent democratic processes in society and contribute to opinion polarization and fake news dissemination. Personalization algorithms might significantly influence opinion dynamics processes because they control information flows between individuals. Relatively recently, scholars began to combine ideas of personalization algorithms with opinion formation models^54–56^.

Review studies on opinion formation models emphasize that there is an urgent need in testing these models against real data^2,4,5^. OSNs may serve as a potential solution here due to the fact that they can provide well-resolved longitudinal data describing opinion dynamics processes. Of course, this approach comes with some weaknesses^2^. One of them stems from the fact that neighboring vertices in social networks tend to have similar attributes, a phenomenon referred to as homophily. In such settings, without an opportunity to control for confounding factors, it is sometimes extremely difficult to disentangle social influence from, for example, selectivity or the presence of common stimuli^47,57–59^. Further, the substantial variety of opinion formation models complicates the situation concerning model validation because each of these models may require a specific data format. As a result, each new dataset on opinion dynamics will potentially have a limited area of application in the sense that it could be investigated only by a restricted number of opinion formation models. The current article attempts to resolve the latter problem by proposing a general opinion formation model that, on the one hand, can be easily calibrated on empirical data, upon which it imposes a relatively small number of requirements and that, on the other hand, is extremely flexible and can approximate a wide variety of the existing micro-influence assumptions and models.

## Methods

### Model

We consider the system of $N$ agents that are connected by a social network $G$ (by default, we assume that it is static). Each agent’s opinion may take one of $m$ values from the set $X=\left\{{x}_{1},\dots ,{x}_{m}\right\}$ that represents a discrete opinion space, a construction that is extensively studied in the sociophysics literature^1^. In some situations, one may assume that a binary relation is predetermined on the opinion space whereby types ${x}_{1}$ and ${x}_{m}$ stand for the most radical and polar position in that space: ${x}_{1}<{x}_{2}<\dots <{x}_{m}.$ Depending on the context, we will endow these quantities with different values.

In our model, the time is discrete; we denote the opinion of agent $i$ at time $t$ by ${o}_{i}\left(t\right)\in X$. The population of agents having opinion ${x}_{k}$ at time $t$ is described by the quantity ${Y}_{k}\left(t\right)\in \left\{\mathrm{0,1},\dots ,N\right\}$:

$${Y}_{k}\left(t\right)=\#\left\{i | {o}_{i}\left(t\right)={x}_{k}\right\},$$

where $\#\left\{\dots \right\}$ denotes the cardinal number of the set.

Note that we assume that the system is “conservative” (agents do not leave the system, and there are no incoming agents): $\sum_{k=1}^{m}{Y}_{k}\left(t\right)=N$ for any $t$.

The dynamics of the model are organized as follows. Starting from some initial opinion vector $${\left[\begin{array}{ccc}{o}_{1}\left(0\right)& \dots & {o}_{N}\left(0\right)\end{array}\right]}^{T}$$, at each time point $t$, a randomly chosen agent $i$ with opinion ${o}_{i}\left(t\right)$ is influenced by one of their neighbors $j$ in social network $G$ (the neighbor is also selected at random). Hence, the opinion of the focal agent (without loss of generality, we assume that ${o}_{i}\left(t\right)={x}_{s}$) changes (or remains the same) according to a special rule. This influence mechanism is asymmetric: the opinion of agent $j$ (let ${o}_{j}\left(t\right)={x}_{l}$) does not change following the interaction. Being influenced by $j$’s opinion, $i$’s one can take one of $m$ possible values from set $X$. In the model, it is assumed that the outcome of the influence event is not defined deterministically (as typically occurs in opinion formation models) but involves randomness represented by distribution $\left\{{p}_{s,l,1},\dots ,{p}_{s,l,m}\right\}$ (whereby $\sum_{k=1}^{m}{p}_{s,l,k}=1$ for any $s$ and $l$) in which quantity ${p}_{s,l,k}$ is the probability that an agent with opinion ${x}_{s}$ will switch their opinion to ${x}_{k}$ after being influenced by an agent holding opinion ${x}_{l}$:

$${p}_{s,l,k}=\mathrm{Pr}\left[{o}_{i}\left(t+1\right)={x}_{k} | {o}_{i}\left(t\right)={x}_{s},{o}_{j}\left(t\right)={x}_{l}\right].$$

As a result of this interaction, if agent $i$ takes opinion, say, ${x}_{w}$, variables ${Y}_{s}$ and ${Y}_{w}$ change accordingly:

$${Y}_{s}\left(t\right)={Y}_{s}\left(t-1\right)-1,{Y}_{w}\left(t\right)={Y}_{w}\left(t-1\right)+1.$$

After that, the next iteration begins, and a new agent is chosen at random, and so forth.

Quantities ${p}_{s,l,k}$ introduced above form a 3-D matrix $P=\left[{p}_{s,l,k}\right]\in {\mathbb{R}}^{m\times m\times m}$ where $s,l,k\in \left\{1,\dots ,m\right\}$, which governs the opinion dynamics that unfold on the social network. This matrix, which we will refer to as the transition matrix hereafter, prescribes the probabilities of opinion shifts. Note that ${p}_{s,l,s}$ represents the probability of staying at the current position after interaction with opinion ${x}_{l}$, whereas ${p}_{s,s,k}$ indicates the likelihood of taking opinion movement ${x}_{s}\to {x}_{k}$ after interaction with an agent espousing the same opinion ${x}_{s}$. In the following, it will be convenient to represent different transition matrices by considering their slices over the first index. We will denote these slices, which are row-stochastic 2-D matrices, by ${P}_{s,:,:}\in {\mathbb{R}}^{m\times m}$. In brief, the matrix ${P}_{s,:,:}$ outlines the behavior of an agent who has opinion ${x}_{s}$. Its rows indicate the opinion of an influence source, and its columns represent potential opinion options (see Example A1 in Appendix for details):

$${P}_{s,:,:}=\left[\begin{array}{ccc}{p}_{s,\mathrm{1,1}}& \dots & {p}_{s,1,m}\\ \dots & \dots & \dots \\ {p}_{s,m,1}& \dots & {p}_{s,m,m}\end{array}\right].$$

The transition matrix encodes the influence processes in the model and may reflect a broad set of effects including the persuasiveness of individuals as a function of their current positions and general trends in opinion change caused by influential external events^31^. For example, political debates or interviews could increase the reputation of a particular person and, thus, strengthen the persuasiveness of arguments in support of this person. Further, the transition matrix should be sensitive to such events and transform as a response to them. The number of parameters in the transition matrix depends only on the number of possible opinion values rather than on the total number of agents.

The model elaborated is extremely general and can capture a broad set of micro-influence mechanisms and models introduced in the literature (see Examples A2–A4 in Appendix for details). Despite the model operating with discrete-type opinions, it can handle micro assumptions introduced for continuous opinions (assimilative influence, bounded confidence, and antagonistic interactions—this ability becomes possible after arranging the discrete opinions ${x}_{1},\dots ,{x}_{m}$ with the binary relation. However, our model’s flexibility is not without limitations. One can notice that the model assumes that agents with similar opinions should act equally on average in similar situations (that is, being exposed to comparable influence opinions), an assumption that significantly reduces the model’s predictive power because not all ties transmit influence on an equal basis, and not all agents are equally influential. In other words, our model can reproduce only the average patterns of opinion formation processes and approximate only anonymized forms of communications (whereby all influence weights are equal). This issue makes the model less flexible than, for example, the DeGroot model, which allows individuals to allocate different influence weights to their peers. On the other hand, our model can easily explain the situation when an agent acts differently (by choosing positive or negative shift) as a response to the same influence opinion. This ability may be (i) due to the model’s stochasticity and (ii) because the model allows encoding of different opinion-changing strategies, depending on the current opinion of the focal individual.

In this paper, we focus on the most simple and atomic communication regime in which only two agents communicate at one iteration. Despite there being other communication regimes, such as many-to-one^22^, many-to-many^41^, and one-to-many^60^ (which is extremely important in the online context), we have decided to elaborate our model upon one-to-one interactions for several reasons. First, as aforementioned, it is the simplest possible communication regime, and many real-world interactions can be modeled using this one. For example, in the online environment, when a user inspects their news feed, they proceed information (posts, comments, etc.) in a sequential manner, post by post, comment by comment. Thus, this process can be decomposed as a sequence of one-to-one communications, each of them representing how a post or comment influences the user. Further, if one wants to model the situation when one actor (say, a blogger) exerts influence on many other users simultaneously (in fact, implementing this form of communication may affect sufficiently the macro outcomes of opinion dynamics^60^), then our model should be modified by transforming how individuals are chosen for interactions, but the interactions themselves remain one-to-one. Besides, scholars have found that deterministic many-to-one communications may be approximated by one-to-one gossip-like interactions^61^. And the final reason is technical—the averaging procedure, which one should apply in the case of the many-to-one regime to approximate the opinion of individuals who exert influence on a focal agent, is quite natural for continuous opinions but is far less convenient for discrete-type opinion spaces.

### Model identification

To calibrate the elaborated model, one needs to know (i) the trajectories of individual opinions and (ii) the history of the individuals’ communications. To be more precise, for a given individual $i$, one must know their opinion ${y}_{i}$ before communication, the opinion of the influence source ${y}_{i\leftarrow }$, and the focal agent’s opinion ${z}_{i}$ after communication. One should first apply the procedure of discretization on experimental opinions if these opinions are initially continuous. Here, one should find the most appropriate discretization step, which should be a sort of compromise: a step that is too large could lead to losing potentially useful information on individuals’ opinion trajectories, whereas too small a step results in a sharp increase in the number of transition matrix elements, which are now highly difficult to interpret and give way to unnecessary data fluctuations. The resulting discrete opinions (for convenience, we denote them similarly) can be used to estimate the transition matrix elements:

$${p}_{s,l,k}=\frac{\#\left\{i | \left({y}_{i}={x}_{s}\right)\&\left({y}_{i\leftarrow }={x}_{l}\right)\&\left({z}_{i}={x}_{k}\right)\right\}}{\#\left\{i\in I | \left({y}_{i}={x}_{s}\right)\&\left({y}_{i\leftarrow }={x}_{l}\right)\right\}}.$$

To put it simply, ${p}_{s,l,k}$ is computed as the fraction of individuals who made opinion change ${x}_{s}\to {x}_{k}$ among those whose opinion is ${x}_{s}$ and are influenced by opinion ${x}_{l}$. To compute all the transition matrix components, one needs to be provided with a substantial opinion diversity, which ensures that all combinations of ${x}_{s}$ and ${x}_{l}$ are represented in the data. Otherwise, the available statistics would be insufficient to calibrate the transition matrix. Individuals’ opinions should be represented at least twice in the data: before and after interactions. However, longer opinion trajectories will be useful because they provide more room for analysis.

Note that we do not impose any requirements on the nature of empirical opinions. They could be discrete—in this case, we will consider low-dimensional transition matrices, or continuous—then, we firstly discretize opinions. In principle, the history of an individual’s communications (with whom they talked), which is highly difficult to retrieve in non-laboratory settings, can be replaced by more simple forms of structures of social connections, such as a friendship network, which could be relatively easily retrieved from the Web. Of course, more detailed information on how individuals interact with each other will make the model more precise, but in the following section, we will demonstrate that even a simple friendship network may serve as a good approximation of the actual communication network in the sense that it could simulate artificial social systems consistent with empirics at the macro scale.

### Mean-field analysis

The model elaborated above can be studied using mean-field approximation. We assume that the social network is a complete graph whereby each agent can communicate with each other.

Establishing scaled time $\tau =\frac{t}{N}$ and introducing normalized quantities ${y}_{f}\left(\tau \right)=\frac{{Y}_{f}\left(\tau \right)}{N}$, for large $N$, we obtain the nonlinear autonomous system of differential equations (see Appendix 3 to inspect the derivation of this system):

1 $$\frac{d{y}_{f}\left(\tau \right)}{d\tau }=\sum_{s=1}^{m}\sum_{l=1}^{m}\sum_{k=1}^{m}{y}_{s}\left(\tau \right){y}_{l}\left(\tau \right){p}_{s,l,k}\left({\delta }_{k,f}-{\delta }_{s,f}\right), f\in \left\{1,\dots ,m\right\}.$$

Because the right side of (1) is a polynomial, we can guarantee that the Cauchy problem for system (1) has a unique solution, which is an analytic function of model parameters ${p}_{s,l,k}$ and initial conditions ${y}_{f}\left(0\right)={y}^{f}$.

### Calibrating the model with empirical data

Let us now exemplify the elaborated analytical results with data describing real opinion dynamics processes. The corresponding dataset was recently reported in Refs.^62,63^, where the author analyzed longitudinal data representing the dynamics of (continuous) opinions of a large-scale sample (~ 1.6 M) of VKontakte (the most popular OSN in Russia that is organized in a very similar manner to Facebook) users in 2018 (hereafter—Dataset). The sample was constructed by randomly chosen individuals among those who meet some natural criteria (see Appendix 5 for details). Users’ opinions were estimated on the interval $\left[\mathrm{0,1}\right]$ using a machine learning methodology (more precisely, a trained logit model was applied to users’ accounts whose followees’ lists were encoded using the one-hot encoding strategy), where extreme positions 0 and 1 represent maximal opposition and support for the current Russian government correspondingly. These estimations are based on information sources (public pages and accounts of famous people such as bloggers or politicians) that are followed by users. This issue reflects the fact that individuals tend to consume information coherent with their current opinions^64^. Dataset includes three opinion snapshots (obtained in February, July, and December 2018) overall. In this regard, changes in users’ opinions indicate adjustments in their followees lists. The second component of Dataset is the social network that represents friendship connections between the sample users in December 2018. This social network is a connected undirected graph because in the preliminary stage, the sample was cleared of isolated subgroups of online friends in such a manner that the resulting social network consists of one (giant) connected component^63^. Note that only 0.7% of all nodes were removed during this procedure. It is also worthy to state that users’ estimated opinions are independent of friendship ties because information sources that were used in the opinion estimation procedure were excluded from the sample. Friendship ties were not used in opinion estimation because this type of connection has a different nature, compared to one-directional ties between ordinary users and information sources: whereas the first ones require the agreement of both sides to appear and likely stem from shared social contexts and a desire to maintain a communication channel, the second type of connections needs only one follow-type click from the user and may easily appear to adapt to the user’s current interests and views (or removed due to the same reasons without hurting the other side)^62^.

Using the first two opinion snapshots from Dataset (see Appendix 6 for details), we obtain the following transition matrix (rounded to 3 decimal places) for case $m=2$ (note that all results presented below remain virtually the same if one uses different snapshot sequences (e.g., first and third or second and third)):

2 $${P}_{1,:,:}=\left[\begin{array}{cc}0.975& 0.025\\ 0.952& 0.048\end{array}\right],{P}_{2,:,:}=\left[\begin{array}{cc}0.066& 0.934\\ 0.049& 0.951\end{array}\right].$$

One can observe that transition matrix (2) is very different from the one that represents the voter model (see Example A2 in Appendix); in the current case, agents rarely change their opinions if they are exposed to challenging positions. In turn, they have a nonzero chance of anticonformity. Nonetheless, the likelihood of a non-static opinion shift slightly increases if two agents with opposite positions communicate, compared to when they have similar opinions. System (1) calibrated with transition matrix (2) (see formula (A3) in Appendix) has only one meaningful (located in the unit square) equilibrium point ${y}_{1}^{*}\approx 0.644,{y}_{2}^{*}\approx 0.356$, which is asymptotically stable. Further, if ${y}_{1}<{y}_{1}^{*}$, then the phase velocity of ${y}_{1}$ is positive (indicating that ${y}_{1}$ will grow in the immediate future) and if ${y}_{1}>{y}_{1}^{*}$, then the phase velocity is negative (${y}_{1}$ will decrease). That is, for every starting point, the system should end up in this equilibrium.

Let us now consider triple opinion space $X=\left\{{x}_{1},{x}_{2},{x}_{3}\right\}$ ($m=3$). In this configuration, opinions ${x}_{1}$ and ${x}_{3}$ stand for antagonistic positions, whereas ${x}_{2}$ is somewhat neutral, located in the center. The transition matrix in this case (obtained similarly to how we calculated transition matrix (2)) takes the following form:

3 $${P}_{1,:,:}=\left[\begin{array}{ccc}0.96& 0.04& 0\\ 0.942& 0.057& 0.001\\ 0.907& 0.091& 0.002\end{array}\right],{P}_{2,:,:}=\left[\begin{array}{ccc}0.039& 0.952& 0.008\\ 0.021& 0.969& 0.01\\ 0.02& 0.944& 0.036\end{array}\right],{P}_{3,:,:}=\left[\begin{array}{ccc}0.001& 0.082& 0.917\\ 0.001& 0.07& 0.929\\ 0.001& 0.054& 0.945\end{array}\right].$$

**Figure 1:**
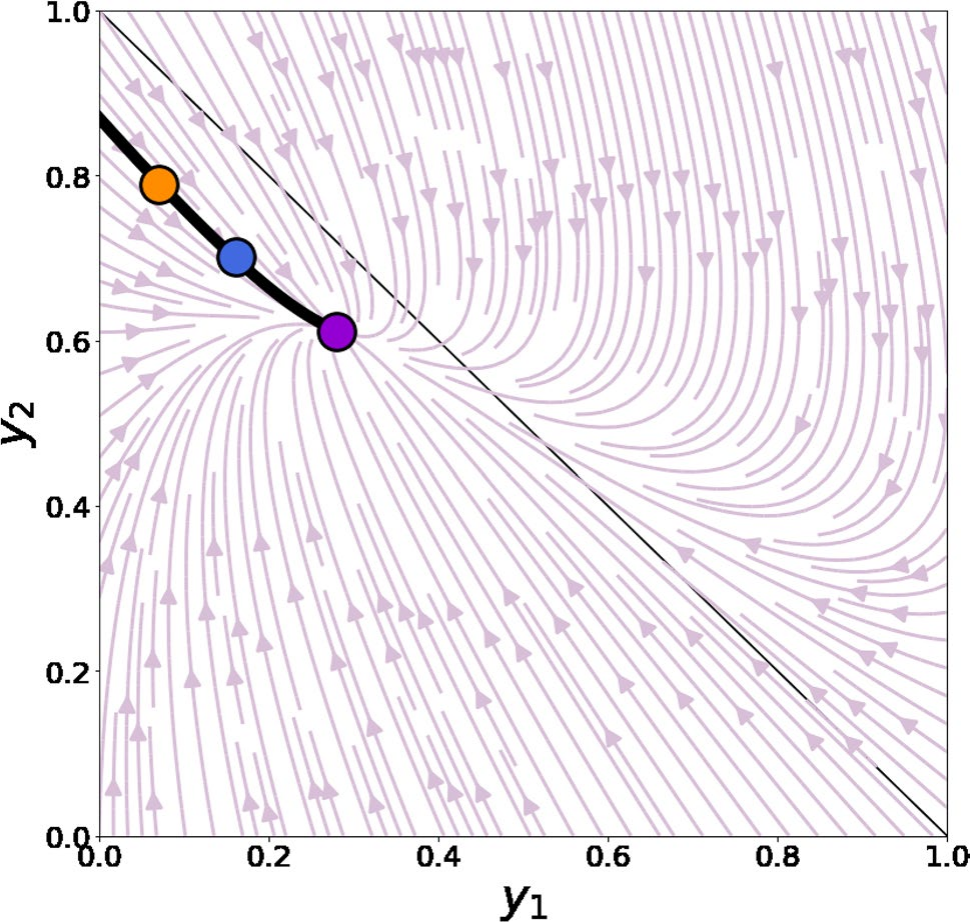
The phase portrait for system (1) calibrated with transition matrix (3). The violet circle marks the equilibrium point. The straight black solid line plots ${y}_{1}+{y}_{2}=1$. We are interested in the area ${y}_{1}+{y}_{2}\le 1$ beneath this line. The phase portrait of the system demonstrates that the equilibrium point is an asymptotically stable nodal sink that attracts the system regardless of its initial point. The blue and orange circles lying on the same phase curve (bold black line) represent opinion distributions ${y}_{1}=0.161,{y}_{2}=0.702,{y}_{3}=0.137$ (see Table 1 in the following subsection) and ${y}_{1}=0.08,{y}_{2}=0.78,{y}_{3}=0.14$ (see (6) in subsection Matching simulations and empirics) correspondingly.

Note that in (3), all components are also rounded to 3 decimal places for demonstrative purposes. In fact, all components in this transition matrix are positive. The slices of transition matrix (3) reflect several remarkable features. First, larger values of opinion difference between individuals increase the rate of positive influence. One can observe this trend by inspecting slices ${P}_{1,:,:}$ and ${P}_{3,:,:}$: for example, the second and third columns in matrix ${P}_{1,:,:}$ are established so that their values increase as the index of the rows also rises. Further, individuals located in the middle of the opinion space may make negative shifts (pointing in the direction opposite to the influence source): ${p}_{\mathrm{2,1},3}>0,{p}_{\mathrm{2,3},1}>0$. However, according to (3), positive shifts (directing towards the source of influence) are more likely than negative ones: ${p}_{\mathrm{2,1},3}<{p}_{\mathrm{2,1},1},{p}_{\mathrm{2,3},1}<{p}_{\mathrm{2,3},3}$. System (1) augmented with transition probabilities (3) has only one meaningful (located in the unit square) equilibrium point that can be obtained graphically. We find that ${y}_{1}^{*}\approx 0.28$, ${y}_{2}^{*}\approx 0.611$ (and ${y}_{3}^{*}\approx 0.109$ correspondingly). The Jacobian matrix at the equilibrium point has two different negative eigenvalues; hence, we can identify the behavior of the phase curves near the equilibrium point, which is an asymptotically stable nodal sink because the Jacobian matrix at this point has two different negative eigenvalues (see Figs. 1 and A1 (in Appendix)).

### Numerical experiments

We perform extensive numerical experiments to investigate the behavior of our model and, more specifically, support the analytical results derived above. Besides, we would like to understand whether our model can generate artificial social systems close (in some respects) to those observed empirically. We recognize that it is unlikely that we will be able to predict the opinion trajectories of specific individuals. However, we hope to predict the system’s dynamics at the macroscopic level.

We build our further analysis on the investigation into how the model works on synthetic random networks by employing the following macroscopic metrics.

#### M1

The fraction of individuals ${y}_{i}$ who have opinion ${x}_{s}$ for $s\in \left\{1,\dots ,m\right\}$. The combination of variables ${y}_{1}\left(t\right),\dots ,{y}_{m}\left(t\right)$ represents public opinion at time $t$. In what follows, we will refer to them as public opinion variables.

#### M2

Assortativity coefficient, which measures whether the system at hand is homophilic. To put it simply, the assortativity coefficient measures how similar the neighboring opinions are, compared with the configuration in which edges are placed at random (see Appendix 8 for details)^65^. For homophilic networks (most empirically observed social networks are homophilic), the assortativity coefficient takes positive values.

#### M3

The dissimilarity coefficient $D$, which effectively assesses the current level of polarization^66^. This measure is defined as the standard deviation of all pairwise opinion distances and takes values in the range between $D=0$ (no polarization—all opinions are equal) and $D=1$ (the highest level of polarization—individuals are divided into two equally sized camps located on the edges of the opinion space), provided that opinions lie in the interval $\left[-1, 1\right]$.

To compute the assortativity coefficient, we use opinion values ${x}_{1}=0,{x}_{2}=1,\dots ,{x}_{m}=m-1$. Instead, if we calculate the dissimilarity coefficient, then we reinitialize these values to make them lie in the interval $\left[-1, 1\right]$: ${x}_{1}=-1,\dots ,{x}_{m}=1$.

In experiments, we consider $N=2000$ agents, who are endowed with randomly generated initial opinions. We tested different initial opinion configurations; however, we found that they have no influence on the model’s asymptotic behavior (see Figure A2 in Appendix and Online Supplementary Materials). Therefore, unless otherwise stated, opinions are initialized from the generalized Bernoulli distribution in which we set the uniform vector of probabilities (${y}_{i}\left(0\right)=1/m$ for $i\in \left\{1,\dots ,m\right\}$—for simplicity, we refer to it as to the uniform distribution). For large $m$, this initial opinion configuration is characterized by $C\approx 0,D\approx 0.5$. In each experiment, a new network is generated, as well as new initial opinion values. Apart from the complete graph model, we employ four synthetic graph models that are widely used in the social simulations literature^54^: (i) Erdős–Rényi network, (ii) random geometric network, (iii) Watts–Strogatz networks (WS1, WS2, and WS3), and (iv) Barabási–Albert network. Detailed information regarding network configurations and properties is presented in Table A2 (Appendix). Experiments typically lasted no more than one million iterations, a time interval that is sufficiently large to inspect the model’s behavior. We repeated each experiment 20 times to gain more precise estimations.

**Table 1:** Values of metrics **M1**–**M3** drawn from dataset.

Metric	Discretization step											
	*m* = 2				*m* = 3					*m* = 10		
Public opinion variables	*y*_1_	0.568	0.576	0.582	*y*_1_		0.161	0.168	0.174			
	*y*_2_	0.432	0.424	0.418	*y*_2_		0.702	0.697	0.692			
					*y*_3_		0.137	0.135	0.134			
Assortativity coefficient	0.109	0.109	0.109		0.106	0.107	0.108			0.141	0.141	0.141
Dissimilarity coefficient					0.575	0.585	0.585			0.356	0.366	0.357

The dissimilarity coefficient in the case of the binary opinion space is not useful and, therefore, we do not calculate it. The dynamics of public opinion variables in the case of the tenfold opinion space are too massive—one can find this information in Ref.^63^ if necessary. Opinion distribution ${y}_{1}=0.161,{y}_{2}=0.702,{y}_{3}=0.137$ that demonstrates public opinion in the first opinion snapshot is presented in Fig. 1 (as the blue circle).

**Figure 2:**
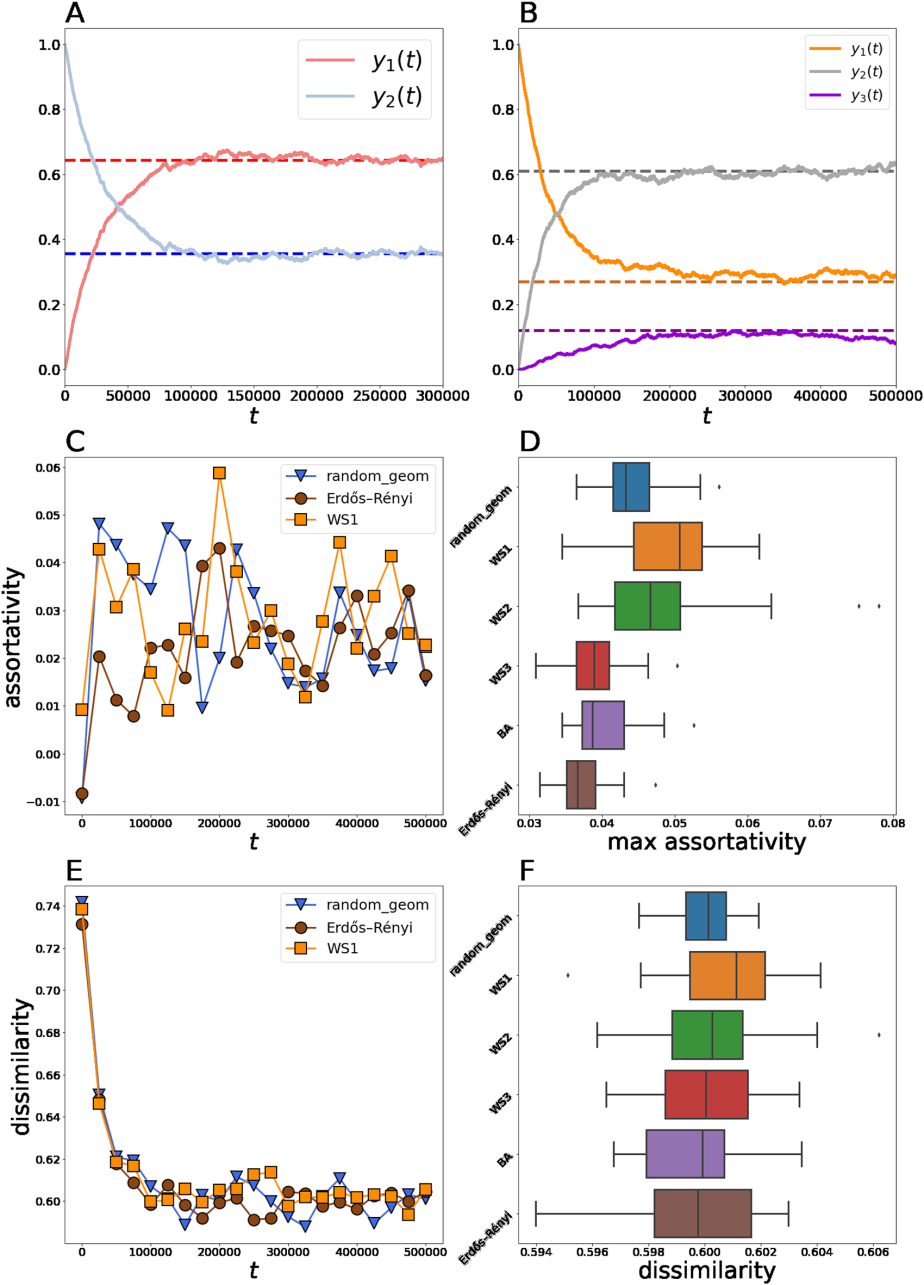
Ideal–typical evolution of public opinion variables in the model in the case of binary (**A**, initial opinions are drawn from the uniform distribution, random geometric topology) and triple (**B**, initial opinions are equal to ${x}_{3}$, BA topology) opinion spaces. Dashed lines plot theoretically predicted equilibrium values. (**C**). Ideal–typical behavior of the assortativity coefficient in experiments. The network topology with no clustering (Erdős–Rényi) reaches lower assortativity values than clustered ones (random geometric and WS1). (**D**). The panel plots how the maximal value of assortativity during a single experiment varies with the network topology. (**E**). Ideal–typical dynamics of the polarization coefficient for different network topologies. (**F**). The limiting value of the dissimilarity coefficient as a function of the network topology. Panels (**C**–**F**) represent experiments for $m=3$.

We estimate the transition matrix using information from Dataset and concentrate on the first two opinion snapshots. We analyze cases $m=2$, $m=3$, and $m=10$. The first two opinion space configurations require only a small number of variables to be parametrized, and this ability is useful in interpretations and for demonstrative purposes. Instead, the tenfold opinion space provides a more precise approximation of the underlying social processes. A further increase in $m$ may lead to unnecessary fluctuations in the data and a sharp increase in the number of transition matric elements. Transition matrices for binary and triple opinion spaces have already been introduced in (2) and (3). The tenfold transition matrix is partially presented (and discussed) in Appendix (Tables A3–A5); its full representation can be found in Online Supplementary Materials.

The immediate observation that could be made from the estimated transition matrices is that the system has no stable states at the microscopic level: for every opinion vector, there is a nonzero probability that a randomly chosen agent will change their opinion at the next time point (even after exposure to the same opinion). However, in the following, we will demonstrate that the system has a stable converging tendency from the perspective of the macroscopic metrics.

Refs.^62,63^ reported that the social system from Dataset is homophilic with the assortativity coefficient of approximately 0.14, which may be considered as a not particularly strong (but noticeable) rate of homophily. Refs.^62,63^ also observed that users’ opinions tend to stretch out to the edges of the opinion space in such a way that the fraction of individuals having middle-located (moderate) opinions decreases, individuals disposed on the left edge grow in number, and right-opinion users tend to maintain their number or slightly decrease. To gain a more systematic understanding of the system, we calculate the metrics **M1**–**M3** using different discretization strategies for all three opinion snapshots from Dataset (see Table 1). We observe the following dynamical patterns: (i) growth of the population of individuals espousing left-side opinions, decrease of those who hold a middle-side opinion, and a relatively small decrease of right-side opinion persons; (ii) extremely small increasing trend in the homophily level; (iii) extremely small increasing trend in the polarization rate. As such, we hypothesize that the model calibrated on the same data should be able to achieve similar metric values (hereafter—reference values) at some point of its evolution and, further, demonstrate the same dynamics patterns near this point.

## Results

### Macroscopic behavior of the model

Our experiments reveal that regardless of the network topology, the behavior of public opinion variables remains the same. The evolution of the model can be decomposed into two periods (see Fig. 2, panels A, B). In the first one, populations of camps ${y}_{1}\left(t\right),\dots ,{y}_{m}\left(t\right)$ nearly monotonically converge to the theoretical predictions ${y}_{1}^{*},\dots ,{y}_{m}^{*}$. In the next period, the system fluctuates around these limiting values. At the beginning, the system is characterized by the almost zero assortativity because opinions are endowed at random. After a simulation has been initiated, the system rapidly becomes homophilic and then features fluctuations in a positive area, demonstrating a relatively low rate of homophily (see Fig. 2, panel C). For example, for the binary opinion space, the maximal assortativity rate observed is 0.05 (obtained under WS1 topology), a value that is far from the empirical reference value (0.109). A similar difference in assortativity values was discerned in the case of the triple (0.078 in simulations against reference value 0.106) and tenfold (0.072 in simulations against reference value 0.141) opinion spaces. Further, we found that more clustered networks (random geometric, WS1, and WS2) tend to produce more homophilic systems (see Fig. 2, panel D). The typical behavior of the polarization coefficient can be easily predicted because we know how public opinion evolves: starting from some point (that is determined by the initial opinion distribution), the polarization coefficient should firstly drift to the limiting value that characterizes the polarization of the stationary state opinion distribution ${y}_{1}^{*},\dots ,{y}_{m}^{*}$. Depending on the initial opinion configuration, this stage of evolution may feature growth (if the initial polarization level is lower than the limiting value) or decrease (if the initial polarization rate exceeds the limiting one). After the limiting value is achieved, the polarization coefficient should fluctuate around it. Further, this limiting value should not depend on the network topology because the latter does not affect the stationary state opinion distribution. Our numerical experiments (see Fig. 2, panels E, F) confirm this proposal. We do not observe any relation between the network topology and the system’s asymptotic polarization.

The presented results indicate that the elaborated model demonstrates a stable converging tendency: its macroscopic parameters tend to some limiting values at first and then feature oscillations around them. Depending on a particular macroscopic metric, corresponding limiting values may (**M2**) or may not (**M1**, **M3**) be affected by the network topology. However, all of them are not sensitive to the initial opinion configuration. These findings contradict Refs.^25,67^, who reported that the community organization of the network is one of the key factors of polarization. Instead, we found that this organization makes the network more homophilic. In the limit $t\to \infty$, the system features opinion fragmentation, which is characterized by persistent disagreement between agents. Further, one can also notice opinion polarization if the system begins from a densely concentrated opinion distribution. For example, if opinions are concentrated near the center of the opinion space, in this case, at the initial stage of the system’s evolution, opinions will be prone to antagonistic or anticonformity-based interactions (in Ref.^33^, this phenomenon was attributed to the striving for uniqueness^68^) and, thus, will move towards the extreme opinion values ${x}_{1}$ and ${x}_{m}$. However, increasing pairwise distances between agents’ opinions will give way to assimilative interactions that are less likely to occur if opinions are too close. This process will continue until a sort of balance between assimilative and antagonistic interactions is reached.

The model exhibits good predictability from the perspective of the public opinion dynamics: the mean-field approximation is plausible not only for complete graphs (under the assumption of which this approximation was obtained) but also in other settings whereby social connections may have a quite complex structure. One can hypothesize that the randomized opinion dynamics protocol presented in the model in general and in the estimated transition matrices in particular is the reason why the model’s predictions regarding the public opinion variables are robust against the network topology (however, it would not be correct to say that the model is insensitive to the structure of the underlying social network: Fig. 2 clearly indicates that the topology has some effect on the level of homophily in the system). Below, we will demonstrate that removing randomness may significantly alter the model behavior and make the mean-field approximation no longer suitable. Previous studies suggest that randomness can sufficiently change macro outcomes and, in some cases, adding randomness into the model can improve the model’s macropredictions^69^. In our case, randomness may be considered as a sort of compromise between micro and macro forecasts; whereas stochasticity suppresses the ability to predict model behavior at the microscopic level, it, apparently, gives the opportunity to achieve agreement between predictions and simulations at the macroscopic level.

Next, the model can reproduce positive assortativity (i.e., can create a homophilic social system). However, the observed homophily rates are far lower than the empirical reference values. Further, dissimilarity demonstrated by the system after stabilization (which is slightly more than the corresponding reference values) provides a clue that the system can reach the reference polarization level if its initial degree of polarization is lower than the asymptotic one—this situation could arise if initial opinions are densely concentrated (for example, near the center of the opinion space).

These findings indicate that we cannot reach the desired level of homophily unless the system is already homophilic prior to the beginning of a simulation run. In this case, however, the assortativity coefficient will decrease until it reaches the asymptotic value (see Appendix 12 for details), and at the reference point, one will observe a decreasing trend. These dynamical patterns contradict what we observe in empirics, and the following question, therefore, arises: What modifications should one add to the model to make it possible to reproduce empirically observed patterns?

### Explaining discord between empirics and simulations

Let us present some possible explanations of the observed discord in the values of the assortativity coefficient between simulations and empirics and feasible avenues for resolving this conflict.

### Measurement errors in dataset

A discerned divergence between numerical experiments and empirics may stem from methodological errors in obtaining the underlying empirical data. Because these empirics were derived through a natural experiment in which some heuristics were implemented (recall that it was assumed that users’ opinions are reflected by the information sources they follow, the social network was constructed based on online friendship ties ignoring other possible communication channels, and the influence opinion directed on a user was computed as the average of opinions of the user’s friends), it could mean that opinions and influence weights were obtained with some errors. As such, (i) the empirical reference values we strive to equal may be incorrect, (ii) transition matrices may be estimated with errors. The latter issue could lead to changes in the model’s predictions. A slightly different idea is to suppose that individuals’ opinions, as well as the social network, were estimated faithfully, but the transition matrix is identified with errors. In this case, we can rely on Table 1 but still should treat the estimated transition matrices with caution.

Let us assume that empirical reference values we strive to be equal to are estimated correctly, and the problem is rooted in errors in the estimated transition matrix only. The intuition behind this assumption is that the estimation of reference values is a less complex (but not an easy!) task than the transition matrix identification: to obtain reference values, one should have only users’ opinions and the structure of the social network, whereas additional problems could arise while estimating the transition matrix. First, some ties may be more successful in social influence transmission^70^. Besides, an influence system retrieved from the OSN is likely to be incomplete because it neglects to consider the influence beyond this OSN. Third, one more effect may stem from our algorithm of the transition matrix identification and the nature of data: opinion dynamics presented in Dataset are identified under the assumption of many-to-one interactions, whereas the model assumes one-to-one interactions. Further, such factors as selectivity or personalization algorithms (see below) may dominate. And finally, Dataset is built under the assumption that friendship connections (retrieved together with the second opinion snapshot) are static. As a result, the real transition matrices may differ from the estimated ones.

Let us now concentrate on how possible errors in the estimated transition matrices may affect the model’s behavior and its predictions. To address this problem, we consider the binary opinion space, in which these mistakes may be parametrized by just four variables implemented in transition matrix (2). Because the dissimilarity coefficient is meaningless in the case of the binary opinion space, we focus on metrics **M1** and **M2**. We parametrize errors in transition matrices as follows:

4 $${P}_{1,:,:}=\left[\begin{array}{cc}0.975+\alpha & 0.025-\alpha \\ 0.952-\beta & 0.048+\beta \end{array}\right],{P}_{2,:,:}=\left[\begin{array}{cc}0.066+\varphi & 0.934-\varphi \\ 0.049-\psi & 0.951+\psi \end{array}\right].$$

In (4), variables $\alpha ,\beta ,\varphi ,\psi$ represent small perturbations in the estimated transition matrix. We focus on three stylized examples:

#### Scenario 1

We fix $\beta =\varphi =0$ and vary $\alpha$ and $\psi$ in the following domain:

$${D}_{1}=\left\{0\le \alpha \le 0.025, 0\le \psi \le 0.049\right\}.$$

By doing so, we range the levels of anticonformity $0.025-\alpha$ and $0.049-\psi$, and in the extreme case $\alpha =0.025,\psi =0.049$ anticonformity is completely suppressed from the model, as well as the stochasticity in the first and second rows of slices ${P}_{1,:,:}$ and ${P}_{2,:,:}$ correspondingly.

#### Scenario 2

We fix $\alpha =0.025,\psi =0.049,\varphi =0$ and vary $\beta$ as follows:

$${D}_{2}=\left\{0\le \beta \le 0.018\right\}.$$

As a result, we investigate how the model behaves under the assumption that there is no anticonformity across different values of $\beta$, which controls for how “persuasive” agents espousing opinion ${x}_{2}$ are. The larger $\beta$ is, the more influential supporters of the right position are. As long as $\beta <0.018$, left-side agents have priority over the right-side ones. If $\beta =0.018$, then we come to the situation when all agents are equally influential regardless of their opinions.

#### Scenario 3

We set $\alpha =\psi ,\beta =\varphi$ and investigate domain

$${D}_{3}=\left\{-0.02\le \alpha \le 0.02, -0.02\le \beta \le 0.02\right\}.$$

Within this domain, all components of (4) are positive, indicating that each opinion shift is possible with some probability. Note that assuming $\alpha =0$ and $\beta =0$, we return to the earlier-obtained transition matrix (2).

Note that in Scenarios 1 and 2, values 0.025, 0.049, and 0.018 are written only for demonstrative purposes. If fact, we use slightly different numbers because transition matrices (2) and (3) are presented after rounding to 3 decimal places.

Let us now describe how the mean-field predictions appear in Scenarios 1–3. In Scenario 1, as long as $\psi <0.049$ (that is, while randomness is presented regarding the anticonformity-type movements for right-side individuals), the mean-field approximation predicts strictly one (asymptotically stable) equilibrium point located in the unit square towards which the system should evolve regardless of the initial point. If, however, $\psi$ reaches the value of 0.049, then one more equilibrium point ${y}_{1}^{*}=0,{y}_{2}^{*}=1$ (the right-side consensus) appears. However, this additional stationary state is always Lyapunov unstable, and the system should ignore it and move towards the first equilibrium even if starts close to the point ${y}_{1}^{*}=0,{y}_{2}^{*}=1$. Further, if $\psi <0.049$ and, additionally $\alpha =0.025$, then the attractor turns out to be the left-opinion consensus ${y}_{1}^{*}=1,{y}_{2}^{*}=0$. For the extreme case $\alpha =0.025,\psi =0.049$, we obtain two polar consensus-type equilibria ${y}_{1}^{*}=1,{y}_{2}^{*}=0$ (Lyapunov unstable) and ${y}_{1}^{*}=0,{y}_{2}^{*}=1$ (asymptotically stable). In this case, if ${0<y}_{1}<1$, then ${\dot{y}}_{1}>0$, indicating that for every starting distribution excepting the right-side consensus, the system should move towards the left-side consensus. Otherwise, the system should stay at the initial point. Note that according to the mean-field prediction, within domain ${D}_{1}$, the system may reach a consensus state if and only if we suppress the possibility of anticonformity-type movements ($\psi =0.049$ or $\alpha =0.025$). Otherwise, the system should end up in a fragmented opinion distribution.

Mean-field predictions in the case of Scenario 2 are characterized as follows. If $\beta <0.018$, then two equilibria ${y}_{1}^{*}=1,{y}_{2}^{*}=0$ (Lyapunov unstable) and ${y}_{1}^{*}=0,{y}_{2}^{*}=1$ (asymptotically stable) coexist, and because the phase velocity ${\dot{y}}_{1}$ is greater than zero if $0<{y}_{1}<1$, it means that starting from any distribution such that ${y}_{1}>0$, one should end up in the equilibrium point ${y}_{1}^{*}=1,{y}_{2}^{*}=0$. However, if $\beta =0.018$, then Eq. (1) predicts that ${\dot{y}}_{1}=0,{\dot{y}}_{2}=0$, indicating that each point is a stationary state, and populations of competing opinion camps should, therefore, be the same as that of their starting values. However, these predictions do not agree with the model phenomenology: in such settings, both points ${y}_{1}^{*}=1,{y}_{2}^{*}=0$ and ${y}_{1}^{*}=0,{y}_{2}^{*}=1$ appear to be equilibria due to the lack of anticonformity.

Scenario 3 is a less complex situation because for each combination of $\alpha$ and $\beta$, the scenario is characterized by the unique global attractor that predicts opinion fragmentation.

However, the mean-field approximation cannot answer how possible errors in the estimated transition matrices may affect the assortativity coefficient. Simulation experiments should shed light on this problem.

### Selectivity

is a well-documented tendency of social actors that we could not ignore, creating ties with those having similar opinions and breaking connections that promote uncomfortable information^44,45^. Along with (assimilative) social influence, selectivity is considered to be a main driver that makes social networks homophilic^48,58^. As such, we may hypothesize that by adding selectivity into the model, we will increase the level of homophily, which is one of our purposes^46,47^.

We incorporate selectivity into our model by introducing parameter $\gamma \in \left[\mathrm{0,1}\right]$ (selectivity rate), which operates as follows. At each time point $\tau$, we select an agent $i$ (with opinion ${o}_{i}\left(t\right)$) and one of their neighbors $j$ (having opinion ${o}_{j}\left(t\right)$) at random. If their opinions are not too distant ($\left|{o}_{i}\left(t\right)-{o}_{i}\left(t\right)\right|\le\Delta o$), then they follow the standard opinion dynamics protocol: agent $i$ changes their opinion in accordance with the transition matrix. Otherwise (if $\left|{o}_{i}\left(t\right)-{o}_{i}\left(t\right)\right|>\Delta o$), with probability $\gamma$ tie $\left(i,j\right)$ is deleted and a new tie appears: agent $i$ creates a connection with a random (not neighboring) vertex $k$ whose opinion lies within interval $\left[{o}_{i}\left(t\right)-\Delta o,{o}_{i}\left(t\right)+\Delta o\right]$. If there are no such vertices (a situation that may arise, for example, if the focal agent is the last representative of the opinion camp), then tie $\left(i,j\right)$ does not disappear (nothing happens). In the contrary case (with probability $1-\gamma$), the standard opinion dynamics protocol is implemented. Thus, at each iteration, either an opinion changes, or the network evolves, or nothing happens. Note that the number of ties in the evolving social network remains the same.

### Personalization systems

are a signature of the contemporary social networking services^52^. Personalization algorithms may significantly influence the macroscopic behavior of opinion dynamics processes because they control information flows between individuals. Relatively recently, scholars began to combine ideas of personalization algorithms with opinion formation models^54,55^. Their results indicate that personalization may both amplify and reduce polarization, subject to the underlying opinion formation model. Further, scholars argue that personalization algorithms may amplify the formation of echo chambers and, thus, increase the level of homophily. Besides, it is unlikely that the opinion dynamics presented in Dataset were not affected by personalization algorithms. On this basis, we need to incorporate personalization in our model. For our purposes, we employ one of the simplest approaches whereby communications between individuals may be declined. More precisely, selected agents $i$ and $j$ do not communicate (and the system goes to the next time step) with probability $\delta$ (personalization rate) if their opinions differ for more than $\Delta o$.

### Implementation details

We implement assortativity and personalization into the model only in the cases of the triple and tenfold opinion spaces. Importantly, we combine them: if selectivity and personalization rates are positive at once, then agents can both change their connections and be affected by the personalization algorithm, as occurs in the following fashion. First, the personalization algorithm checks whether or not $\left|{o}_{i}\left(\tau \right)-{o}_{i}\left(\tau \right)\right|$ is greater than $\Delta o$. If the latter is true, then communication between agents is allowed, and $i$ changes their opinion as usual. Otherwise (if opinions are too distant), the personalization algorithm activates and prohibits the communication with probability $\delta$, and nothing happens (the system goes to the next time step). In contrast, with probability $1-\delta$ communication between agents $i$ and $j$ is allowed. This permitted communication can go in two different ways. In the first one (that occurs with probability $\gamma$) agent $i$ decides to renew their social environment by replacing tie $\left(i,j\right)$ because it makes agent $i$ uncomfortable. The second direction implies that agent $i$ accepts influence from agent $j$ and follows the standard opinion dynamics protocol (whatever it leads to). Note that if we set $\gamma =0$ and $\delta =0$ in the resulting model (Model 2), we return to the previously elaborated model (Model 1).

Model 2 is investigated only under the empirically calibrated transition matrices derived for triple and tenfold opinion spaces. This approach gives us the opportunity to analyze the effects of selectivity and personalization factors on the dynamics of homophily and polarization. In Model 2, we use threshold $\Delta o=0$ if $m=3$ (which is applied to opinion values ${x}_{1}=0,{x}_{2}=1,{x}_{3}=2$) and threshold $\Delta o=3$ if $m=10$ (which is applied to opinion values ${x}_{1}=0,\dots ,{x}_{10}=9$).

We recognize that the implementation of selectivity and personalization affects not only the opinion dynamics itself but also influences the transition matrices that we estimate (for example, transition matrices estimated from Dataset). From this perspective, the most faithful approach would be to use the (ideal) transition matrix, which, combined with selectivity and personalization, will produce (in simulations) those social dynamics by estimating which we will come to the transition matrices estimated from empirics. However, for the sake of simplicity, we build our analysis upon the estimated transition matrices, assuming that these matrices do not depend on selectivity and personalization factors.

### The effect of the uncertainty of transition matrices estimation on the model behavior

**Figure 3:**
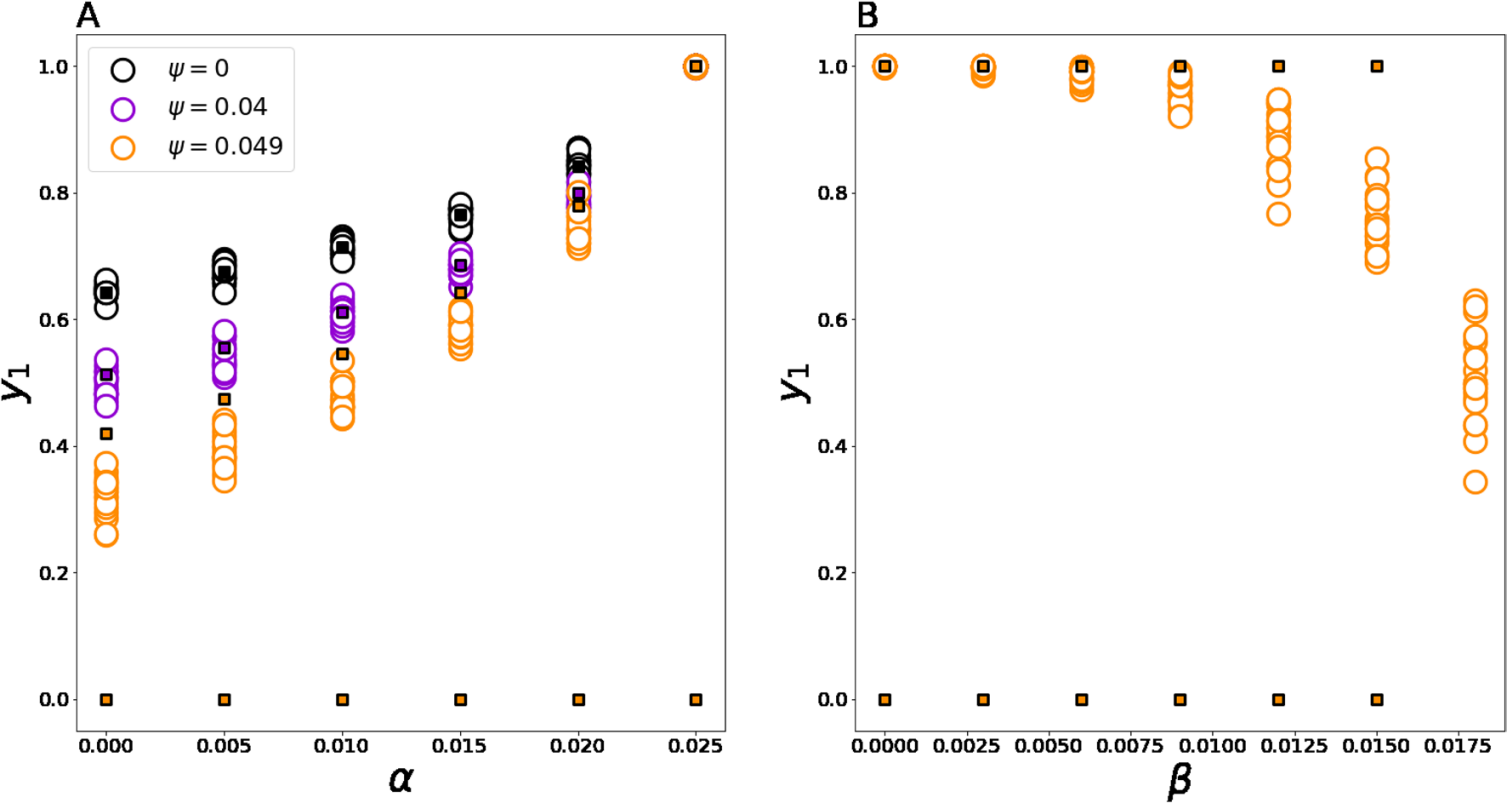
The panels compare mean-field predictions of the model behavior (colored squares) against simulations (white circles) in Scenarios 1 (panel A) and 2 (panels B). In the presented experiments, 2000 agents were considered for which opinions were initialized from the uniform distribution. Limiting opinion distributions were obtained after 1,000,000 iterations.

Our results indicate that the mean-field predictions are plausible in Scenario 1 as long as $\psi$ is less than 0.049 and the number of agents is sufficiently high (see Fig. 3). If, however, $\psi$ is near 0.049 (little to no anticonformity in behavior of individuals espousing right-side opinions), then, under certain topologies, the model tends to deviate from the predicted stationary states by shifting towards the point ${y}_{1}^{*}=0,{y}_{2}^{*}=1$ (which is the Lyapunov unstable equilibrium for $\psi =0.049$). This discord disappears on complete graphs (see Figure A9 in Appendix). On low-clustered networks (Erdős–Rényi, WS3, and BA), such discord is less pronounced (see Figures A6–A8 in Appendix). For other topologies, deviations are more evident. For a fixed topology, deviations between the mean-field predictions and simulations are particularly sound when (i) the number of agents in the system is small and (ii) simulations start near ${y}_{1}^{*}=0,{y}_{2}^{*}=1$ (see Figure A4 in Appendix, panels C–F) because small systems feature larger oscillations and, thus, have more opportunities to fall into this equilibrium. If conditions (i) and (ii) are not satisfied, then the deviations are suppressed while one increases the value of $\alpha$, and in the limiting case $\alpha =0.025$, the system strives to the predicted equilibrium ${y}_{1}^{*}=1,{y}_{2}^{*}=0$. If, however, conditions (i) and (ii) are true, then even for $\alpha =0.025$ the system may avoid ${y}_{1}^{*}=1,{y}_{2}^{*}=0$ and fall into the unstable equilibrium ${y}_{1}^{*}=0,{y}_{2}^{*}=1$ (see Figure A4 in Appendix, panel C). In such settings, the more iterations have passed, the more chances that the system ends up in ${y}_{1}^{*}=0,{y}_{2}^{*}=1$ (see Figure A4 in Appendix, panels C, E). If we completely remove anticonformity and increase $\beta$ for fixed $\varphi =0$, then the model tends also to deviate from the mean-field prediction. However, the system cannot reach the unstable equilibrium ${y}_{1}^{*}=0,{y}_{2}^{*}=1$ unless the number of agents becomes too small and the simulations starts near this equilibrium (see Figure A4 in Appendix, panel D). In the latter case, which is characterized by larger fluctuations, the longer experiments tend to end up in consensus states only. In the case of Scenario 3, the system is well predictable, and no deviations between simulations and the mean-field predictions are observed (see Online Supplementary Materials).

From these results, we can conclude that the possibility of right-side agents to make anticonformity-type movements (and corresponding randomness) is a necessary condition for agreement between the mean-field predictions and simulations. Because the solutions of the Cauchy problem formulated for (1) depend smoothly on the model’s parameters, one can conclude that this condition is necessary for the model predictions being robust against the uncertainty in the transition matrix estimation. Further, the nonzero probability of both anticonformity-type movements ensures that the model will feature opinion fragmentation.

In principle, one can simply attribute anticonformity-type opinion movements or some other, say, the most improbable movement types (appearing in higher-dimensional transition matrices) to measurement errors and, further, remove them from the transition matrix as being inconsistent ones. However, in this situation, three problems arise. On the one hand, our previous analysis demonstrates that by removing such movements, one could significantly alter the model’s predictions. On the other hand, it could also be the case that such “impossible” movements do really happen or, say, are caused by some unobserved effects. And finally, the question of what opinion movements should be considered as improbable ones still matters. For example, in the case of the triple opinion space, one can suppose that those opinion shifts described by components ${p}_{\mathrm{1,1},3}$ and ${p}_{\mathrm{3,3},1}$ (that is, the situations when radical agents change their opinions to opposite ones following communications with like-minded peers) are untenable. We report that if one nullifies in (3) these components, then the limiting values of public opinion variables (${y}_{1}^{*}\approx 0.287$, ${y}_{2}^{*}\approx 0.605$, ${y}_{3}^{*}\approx 0.108$) do not differ significantly from what the undisturbed transition matrix (3) leads to (${y}_{1}^{*}\approx 0.269$, ${y}_{2}^{*}\approx 0.611$, and ${y}_{3}^{*}\approx 0.12$).

Our analysis reveals that for a given network topology, the maximal value of assortativity (which is meaningless if the network is in a consensus state) depends smoothly and positively on errors in (4) excepting the situations when the system reaches a consensus (see Figure A10 in Appendix). This result is intuitively clear. On the one hand, by increasing $\alpha$ or $\psi$, we reduce the likelihood that like-minded agents will have different opinions after an interaction. On the other hand, higher values of $\beta$ or $\varphi$ amplify the probability of opinion adoption; thus, neighboring agents with initially different positions are more likely to espouse similar ones after interaction.

We report that within Scenario 3, the minimal disturbance (in the Euclidean metric) we should make with the transition matrix to achieve the acceptable value of the assortativity coefficient is $\alpha =0.02$ and $\beta =0$ (in the case of highly clustered networks). The resulting transition matrix

$${P}_{1,:,:}=\left[\begin{array}{cc}0.995& 0.005\\ 0.952& 0.048\end{array}\right],{P}_{2,:,:}=\left[\begin{array}{cc}0.066& 0.934\\ 0.029& 0.931\end{array}\right]$$

will be able to produce a sufficiently homophilic social system. However, this estimation does not work for low-clustered networks, for which one should take a more disturbed transition matrix (by establishing higher values of $\alpha$ and $\beta$—see Figure A11 in Appendix).

### Macroscopic behavior of model 2

**Figure 4:**
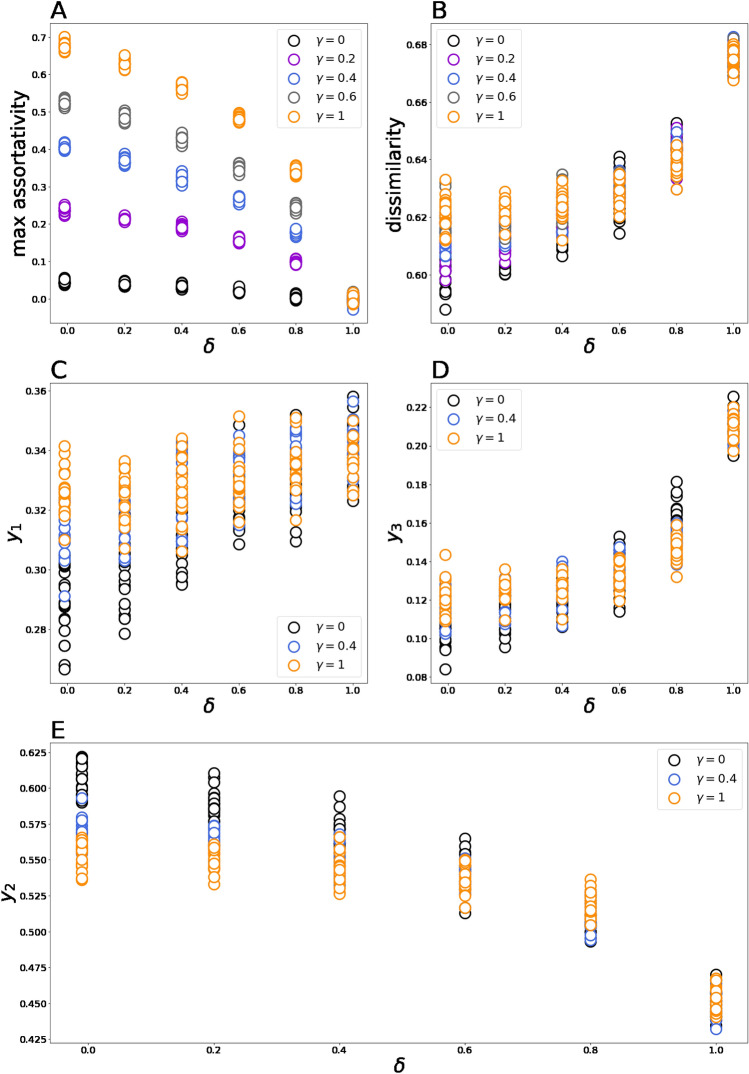
Panels plot how the limiting values of macroscopic metrics **M1**–**M3** depend on the personalization level ($\delta$) and selectivity rate ($\gamma$). The results presented in this Figure were obtained on random geometric networks in the triple opinion space.

The presence of selectivity and personalization does not alter the system’s qualitative behavior (such behavior is inherited from Model 1). Besides, we have observed no situations when the social network becomes disconnected. Nonetheless, we notice that selectivity and personalization affect the limiting values of the macroscopic metrics (see Fig. 4). Figure 4 indicates that higher selectivity rate values lead to more homophilic systems, as expected. However, personalization has the opposite effect on assortativity. The latter effect is explained as follows. If one increases the level of personalization, then the chance of communication between individuals espousing sufficiently different positions is reduced. In this case, negative movements, which reduce the level of homophily and, at the same time, increase polarization, tend to proliferate. Note that for large personalization rate values, the effect of selectivity is reduced. In limiting case $\delta \to 1$, we obtain the system characterized by the zero level of assortativity regardless of the selectivity rate. In this case, only sufficiently similar opinions can interact; thus, all these opinions can do is make antagonistic interactions. The most homophilic system is obtained if $\gamma =1$ and there is no personalization; then, we obtain $C\approx 0.7$. Interestingly, the effect of topology observed for Model 1 (more clustered networks produce more homophilic systems) disappears when we increase the selectivity or personalization rates (see Figure A12 in Appendix). Both personalization and selectivity (as long as personalization is not too high) have a positive effect on the system’s polarization. However, in contrast to the assortativity coefficient, the dissimilarity coefficient varies in relatively small intervals $\approx \left[0.6, 0.68\right]$. In limiting case $\delta \to 1$, the dissimilarity coefficient fluctuates around the value $D\approx 0.675$ and does not depend on the selectivity rate. The limiting values of public opinion variables do also vary with the personalization and selectivity rates. As long as $\delta$ is not very high, the selectivity rate has a positive effect on the populations of left- and right-opinion individuals (for center-opinion agents, the effect is opposite). For large $\delta$, this effect tends to disappear (or even become inverse—as for right-opinion individuals). If $\delta$ takes the highest value possible, then the selectivity factor has no influence on the limiting values of public opinion variables. The personalization rate has a clear positive effect on the number of right-opinion individuals. In contrast, larger values of $\delta$ tend to reduce the limiting value of ${y}_{2}$. We also observe a not very sound positive relation between $\delta$ and ${y}_{1}$. To conclude, both left- and right-opinion agents tend to gain from introducing selectivity and personalization, but right-opinion ones do it more (however, these opinions are still in the minority at the equilibrium).

### Matching simulations and empirics

Endeavoring to achieve agreement between simulations and empirics, we decided to use the triple opinion space because it is the smallest space in which the notion of opinion polarization makes sense. In this regard, our results considering the effect of errors in the estimated transition matrices (obtained in the case of the binary opinion space) are useless; hence, we establish our further analysis upon Model 2. Based on Fig. 4, we tune parameters $\gamma$ and $\delta$, attempting to get close to the empirical reference values in our simulations. The problem is that we need not only to achieve reference values but, what is important, do that simultaneously. We manage to achieve agreement between simulations and empirics by using two transition matrices in a sequential fashion and establishing selectivity and personalization rates as ${\gamma }^{*}\approx 0.13,{\delta }^{*}\approx 0.15$. In these experiments, we use random geometric graphs as a work-horse topology. The first (“ancient”) transition matrix

5 $${P}_{1,:,:}=\left[\begin{array}{ccc}0.9& 0.1& 0\\ 0.855& 0.143& 0.002\\ 0.768& 0.228& 0.004\end{array}\right],{P}_{2,:,:}=\left[\begin{array}{ccc}0.024& 0.971& 0.005\\ 0.013& 0.981& 0.006\\ 0.012& 0.966& 0.022\end{array}\right],{P}_{3,:,:}=\left[\begin{array}{ccc}0.001& 0.064& 0.935\\ 0.001& 0.054& 0.945\\ 0.001& 0.042& 0.957\end{array}\right]$$

explains how the system achieves opinion distribution

6 $${y}_{1}=0.08,{y}_{2}=0.78,{y}_{3}=0.14,$$

which serves as an initial opinion configuration for the second transition matrix (here, we use data-driven transition matrix (3)). Opinion configuration (6) was found as the point on the phase plane that lies on the same phase curve as the empirically identified reference distribution ${y}_{1}=0.161,{y}_{2}=0.702,{y}_{3}=0.137$ from Table 1 but is located sufficiently earlier in time (see Fig. 1, orange and blue circles). Note that opinion distribution (6) was found at first, and then transition matrix (5) was identified manually (see Online Supplementary Materials) as those for which opinion distribution (6) is an attractor. We recognize that this approach is quite rough because the phase portrait presented in Fig. 1 is no longer suitable once one has added selectivity and personalization into the model. However, the values of ${\gamma }^{*}$ and ${\delta }^{*}$ are not particularly high, and we can assume, therefore, that deviations will not be sound (see Fig. 4). We do not state that there is only one such transition matrix that can lead to opinion distribution (6). In this regard, it would be interesting to investigate the problem of the transition matrix identification for a given equilibrium.

**Figure 5:**
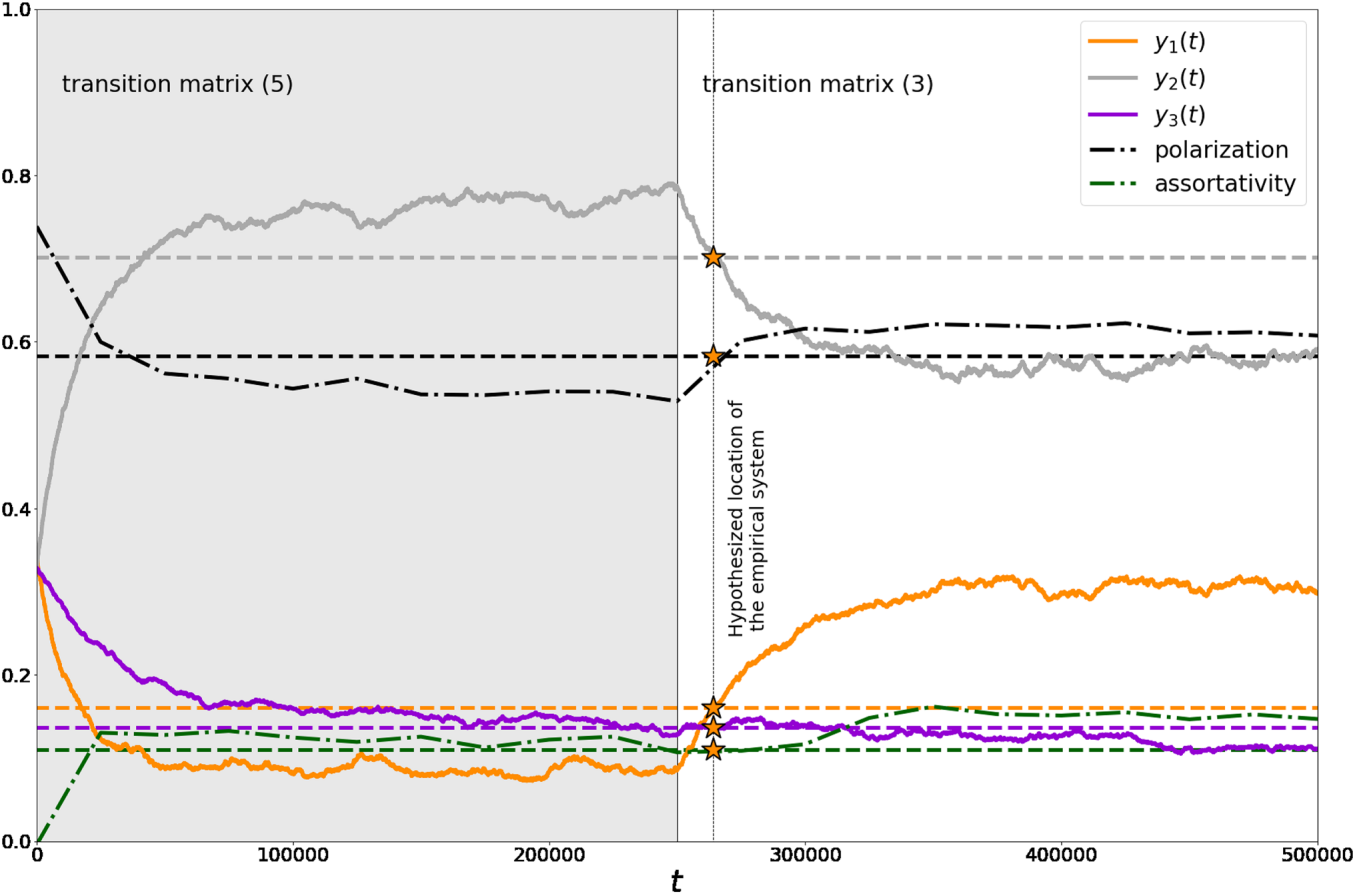
A simulation run for Model 2 if selectivity and personalization are $\gamma =0.13,\delta =0.1$. Dashed horizontal lines plot reference values from Table 1 (we take values from the first opinion snapshot). Common colors represent the same metrics. Initial opinions are drawn from the uniform distribution ${y}_{1}\left(0\right)\approx 0.33,{y}_{2}\left(0\right)\approx 0.33,{y}_{3}\left(0\right)\approx 0.33$. As long as $t<\mathrm{250,000}$, transition matrix (5) is in charge (gray area). At time $t=\mathrm{250,000}$ (the edge between gray and white areas), the system is characterized by opinion distribution (6). From this moment, transition matrix (3) governs the opinion dynamics. At time $t\approx \mathrm{270,000}$ (the dashed vertical line), the macroscopic metrics **M1**–**M3** tend to simultaneously reach reference values (intersections between reference lines and simulation curves are marked roughly with star-like markers). Then, the system moves towards the asymptotic values ${y}_{1}^{*}\approx 0.299,{y}_{2}^{*}\approx 0.591,{y}_{3}^{*}\approx 0.111$ which differ from mean-field predictions due to the presence of selectivity and personalization.

The announced two-matrix simulation run (see Fig. 5) begins from the uniform opinion distribution. At time moment $t=\mathrm{250,000}$, when the system is characterized by opinion distribution (6), transition matrix (5) is replaced by transition matrix (3), and the simulation goes on. At time $t\approx \mathrm{260,000}$, the macroscopic metrics **M1**–**M3** tend to simultaneously reach reference values. Furthermore, the local behavior of the macroscopic metrics near the reference values largely coincides with what we have noticed in the empirical data: the assortativity coefficient increases/fluctuates, the dissimilarity rate increases, as does the fraction of individuals with the left-side opinions, whereas populations of individuals holding other positions (especially the middle-side ones) decrease. This time moment may be understood as the location of the empirical social system in the timeline of the artificially created one. Note that we do not state that there are no more possible approaches to generate an artificial social system that would be consistent with the empirics. However, the advantage of our approach is that we not only reach an agreement between simulations and empirics but also explain how the system was organized before transition matrix (3) takes control over the opinion dynamics. Further, opinion distribution (6) enables to model roughly the dynamics of the public opinion in the Russian Federation just before 2018, including the protest movements in 2017–2018^71,72^ (recall that the empirical data were gathered in 2018)—these events are marked in Fig. 5 as the sharp rise of the fraction of oppositional individuals (${y}_{1}$) in the region $\mathrm{250,000}\le t\le \mathrm{270,000}$.

## Discussion and future work

Selectivity and personalization, which we implemented into the model, sufficiently advanced it and made it more realistic. Hence, we managed to simulate an artificial society that demonstrates properties similar to those observed empirically at the macro scale (at a particular point). Our finding on the (possibly) most appropriate settings that could generate empirically acceptable systems may be employed in several ways. On the one hand, these settings may offer an opportunity to understand how strong personalization and particularly selectivity are in the referenced OSN, from which the empirical data were gathered. More precisely, our results indicate that without selectivity, our artificial systems cannot achieve the desired empirics under the assumption that the transition matrix is estimated correctly. This knowledge can be further used in other studies in which this OSN is involved. However, this idea may be wrong in the case when the transition matrix is estimated with errors. Our current results do not answer the question of whether changes in the transition matrix could lead to full coincidence between simulations and empirics, but they at least hint that they could do. This problem requires additional analysis.

Next, knowing the current state of the system, we can predict its future evolution at the macro scale using the mean-field predictions. According to our analysis (which is, however, limited to the case of the binary opinion space), these predictions, excepting some extreme situations, are robust against the uncertainty of the matrix estimation and the network topology, provided the number of agents in the system is sufficiently high. Unfortunately, only short-term predictions are meaningful because the transition matrix likely changes during long time intervals, reflecting events that occur both in this very OSN or beyond it. To be more specific, we calculate the transition matrix employing the second and third opinion snapshots from Dataset (recall that previous matrices were computed using the first two ones). We obtain the transition matrix (see transition matrix (A7) in Appendix), which slightly differs from its previous version (3). This matrix, implemented in Model 2 (with parameters tuned above), marks the new equilibrium point ${y}_{1}^{*}\approx 0.331,{y}_{2}^{*}\approx 0.546,{y}_{3}^{*}\approx 0.123$, which is a more polarized opinion configuration compared to the one obtained under transition matrix (3). From this perspective, the opinion dynamics of our model can be understood in terms of the transition matrix’s evolution (note that the observed dynamics of the transition matrix may also stem from measurement errors). Our idea of using two transition matrices sequentially in a single experiment stems from the very same concept: we wanted to demonstrate that we observe a long-standing complex social system, and the first (“ancient”) transition matrix explains the previous stage of the system’s life.

The model assumes that all agents are equally influential, and those with similar opinions should act equally on average being exposed to comparable influence opinions. This assumption ignores so-called stubborn agents that are not sensitive to social influence. Depending on the empirical context, such agents could be interpreted differently. For the context considered in this article, it could be online bots. However, in the same context, accounts of political persons or mass media accounts cannot be considered in a similar way because they were already used for opinion estimation, and they should not be understood, therefore, as influence sources. In principle, it is not a problem to add stubborn agents to the model and modify the mean-field equations. Further, one could add one more transition matrix to describe how such stubborn agents influence other, ordinary, agents. As a result, one obtains two transition matrices that include $2{m}^{3}$ elements. To estimate these matrices from online data, one should be able to disentangle ordinary users from bots. These ideas could be extended to account for the fact that even ordinary users may allocate different influence weights on each other depending on users’ characteristics, such as age, gender, or educational level. For example, recent results indicate that common interests or demographics foster the probability of assimilative influence and, thus, reduce opinion polarization^73^. Further, different users may exhibit varying activity patterns on the Web and may also feature diverse persuasiveness in online conversations. These issues could also foster inequality across users regarding the influence they exert on each other. All these ideas could be incorporated into the model in the following fashion. Assume that each agent, in addition to opinion-type (changeable) attribute ${o}_{i}$ taking one of possible $m$ values, is described by attributes ${f}_{i1},\dots ,{f}_{ir}$ (static or also changeable). Let attribute ${f}_{ij}$ may take one of ${m}_{j}$ possible values. In this case, to characterize all possible pairwise interactions, one needs ${\left(m+\sum_{j=1}^{r}{m}_{j}\right)}^{2}m$ values. This modification can inflate the transition matrix and make it difficult to interpret this matrix. On the one hand, it is not a problem if all one requires is predictions. On the other hand, for the given empirical data, this number could be reduced by applying some statistical techniques that will allow to determine the attributes, across which peer influence features the highest variance (or some combinations of attributes—for example, it could be the number of attributes the two communicating agents have in common).

All these ideas constitute a promising direction for future research. In addition, it would be interesting to inspect the model’s behavior for a broader set of transition matrices focusing possibly on analytically accessible low-dimensional cases (within this paper, we focused rather on what happens near the estimated matrices). For example, it would be enlightening to analyze how small perturbations in transition matrices affect the model behavior in the case of the triple opinion space. Another possible direction concerns empirically analyzing how transition matrices appear in other, non-political, contexts and comparing them against each other. Besides, it would be interesting to inspect the transition matrix’s dynamics by considering the evolution of opinions on dynamical networks (such an approach would provide the opportunity to control for the selectivity factor) and determine how external affairs affect these dynamics. Furthermore, an intriguing problem would be to compare the level of selectivity found through the analysis of dynamical networks (that is, directly) against what we obtained in this paper (indirectly).

## Supplementary Information

Supplementary Information.

## Data Availability

Simulation experiments, visualization, and analysis were performed in JupiterHub using Python 3 language. All of the data, codes, and other support information can be found at 10.7910/DVN/NIOEL4 (Online Supplementary Materials).
