# Supplementary material for: A general framework to link theory and empirics in opinion formation models
Source: Sci Rep. 2022 Apr 1;12:5543. doi: 10.1038/s41598-022-09468-3 (PMC8976081; doi:10.1038/s41598-022-09468-3)
Supplement: Supplementary file 1 — Supplementary Information. [file 41598_2022_9468_MOESM1_ESM.docx]

A general framework to link theory and empirics in opinion formation models

Ivan V. Kozitsin

**Appendix 1. Transition matrix organization**

To illustrate the organization of the transition matrix, let us consider the following example.

**Example A1.** Consider the following transition matrix:

$$P_{1,:,:}=\left[ \begin{matrix} 1 & 0 & 0 \\ 0 & 1 & 0 \\ 0 & 0 & 1 \end{matrix} \right],P_{2,:,:}=\left[ \begin{matrix} 0 & 1 & 0 \\ 0 & 1 & 0 \\ 0 & 1 & 0 \end{matrix} \right],P_{3,:,:}=\left[ \begin{matrix} 1/3 & 1/3 & 1/3 \\ 1/3 & 1/3 & 1/3 \\ 1/3 & 1/3 & 1/3 \end{matrix} \right].$$

This transition matrix represents the likelihood of opinion shifts in opinion space $m=3$. According to the transition matrix, an agent who has opinion $x_{1}$ is a conformist who completely follows the opinion of an influence source. In turn, agents who hold position $x_{2}$ are so-called stubborn agents who do not change their opinions in the presence of peer influence. Agents with opinion $x_{3}$ act in a purely random fashion regardless of who influences them.

**Appendix 2. Flexibility of the model**

First, we demonstrate that the classic voter model^1^ can be easily obtained as a special case of our model.

**Example A2.** Let us consider $m=2$*.* To comply with established traditions whereby the binary opinion space is enriched with the spin interpretation, we denote $x_{1}=-1$ and $x_{2}=1$. The following transition matrix

$$P_{1,:}=P_{2,:}=\left[ \begin{matrix} 1 & 0 \\ 0 & 1 \end{matrix} \right]$$

establishes opinion dynamics that reproduce the voter model, in which randomly chosen agents alter their opinions to those of influence sources.

By applying a binary relation to the opinion space (in other words, by introducing geometry in that space), we obtain an opportunity to encapsulate the concepts of bounded confidence and negative influence. We state that an agent makes a positive shift if their opinion moves towards the source of influence. A negative shift occurs if a user’s opinion moves in the opposite direction.

**Example A3.** Let us assume that $m=3$. Consider the following transition matrix slice:

$$P_{2,:,:}=\left[ \begin{matrix} 0.75 & 0 & 0.25 \\ 0 & 1 & 0 \\ 0.25 & 0.25 & 0.5 \end{matrix} \right].$$

In this case, the agent with the middle-side opinion $x_{2}$ may be influenced positively (with probability 0.75) and negatively (with probability 0.25) by opinion $x_{1}$. If influence comes from opinion $x_{3}$, then the focal agent may (i) hold their opinion (with probability 0.25), (ii) make a positive shift (with probability 0.5), and (iii) make a negative shift (with probability 0.25). In turn, if two agents with similar opinions $x_{2}$ communicate, then their opinions do not change.

The notion of bounded confidence is based on the idea that an individual perceives (positive) influence only if the opinion of the influence source is not too far from their own opinion^2^. Bounded confidence may take a strict form—that is, only agents with sufficiently similar opinions may influence each other—or a mild form, whereby agents with different opinions may communicate but with a small probability^3,4^.

**Example A4.** Consider two slices of a transition matrix ($m=4$):

$$P_{1,:,:}=\left[ \begin{matrix} 1 & 0 & 0 & 0 \\ 0.5 & 0.5 & 0 & 0 \\ 1 & 0 & 0 & 0 \\ 1 & 0 & 0 & 0 \end{matrix} \right],P_{4,:,:}=\left[ \begin{matrix} 0 & 0 & 0.2 & 0.8 \\ 0 & 0 & 0.3 & 0.7 \\ 0 & 0 & 0.5 & 0.5 \\ 0 & 0 & 0 & 1 \end{matrix} \right].$$

In this case, agents with opinion $x_{1}$ may be influenced by only those who have the nearest opinion $x_{2}$; they do not accept influence from more distant positions (strict bounded confidence assumption). Instead, agents who occupy the right edge of the opinion space may follow distant positions (by making a one-step opinion shift $x_{4}\to x_{3}$) but with a decreasing rate (mild bounded confidence assumption).

In Example A4, we do not pay attention to so-called leapfrog opinion shifts—situations when an agent’s opinion $x_{s}$ moves towards the opinion of an influence source $x_{l}$, with a magnitude that is higher than the distance between $x_{s}$ and $x_{l}$. In this case, the focal agent’s opinion skips the influence source’s one. Leapfrog opinion shifts are rarely considered in the theoretical studies but nonetheless may be encountered in empirical environments^5–7^. In principle, such situations may be attributed to measurement errors^8^.

Acting in a similar fashion, one could adjust the values of the transition matrix to represent more complex microscopic assumptions on social influence, such as moderated positive influence or combinations of positive and negative influence in which coexistence may take quite nontrivial forms^7,9^.

If the binary relation on the opinion space is introduced, then the limit $m\to\infty$ provides an approximation of a continuous opinion space (without loss of generality, we may consider the interval $\left[ 0,1 \right]$), which has gained substantial attention in the literature^10^.

**Appendix 3. Mean-field approximation**

To derive a mean-field approximation, we assume that the social network is a complete graph whereby each agent can communicate with the other.

Let us first calculate the probability that opinion shift $x_{s}\to x_{k}$ induced by influence from opinion $x_{l}$ will occur at some time moment $t$. This probability can be represented as the product of (i) the probability of selecting an agent with opinion $x_{s}$, (ii) the probability of choosing one of her peers with opinion $x_{l}$, and (iii) probability $p_{s,l,k}$. Because the social network is a complete graph, we estimate probabilities (i) and (ii) as $\frac{Y_{s}\left( t \right)}{N}$ and $\frac{Y_{l}\left( t \right)-\delta_{s,l}}{N}$ correspondingly, where $\delta_{s,l}$ is the Kronecker delta:

$$\delta_{s,l} =\left\{ \begin{aligned} 0, &s\neq l, \\ 1, &s=l. \end{aligned} \right.$$

As a result, we obtain:

$$\Pr\left[ o_{i}\left( t+1 \right)=x_{k},o_{i}\left( t \right)=x_{s},o_{i\leftarrow}\left( t \right)=x_{l} \right]=\frac{Y_{s}\left( t \right)}{N}\frac{Y_{l}\left( t \right)-\delta_{s,l}}{N}p_{s,l,k}. \left( A0 \right)$$

Let us now focus on the probability of the occurrence of the opinion shift that ends on opinion $x_{k}$. To obtain this quantity, one should account for all possible pairwise communications that could end up with opinion $x_{k}$. Technically, it means that one should summarize expression (A0) over $s$ and $l$:

$$\sum_{s=1}^{m} \sum_{l=1}^{m} \frac{Y_{s}\left( t \right)}{N}\frac{Y_{l}\left( t \right)-\delta_{s,l}}{N}P_{s,l,k}.$$

To obtain the probability of the opinion shift that ends on opinion $x_{k}$ but does not begin on opinion $x_{k}$ (i.e., the likelihood that the population of agents holding opinion $x_{k}$ will increase by one, which we denote by $\Pr\left[ Y_{k}\left( t+1 \right)=Y_{k}\left( t \right)+1 \right]$), we should slightly modify the previous expression by adding the factor $1-\delta_{s,k}$ (which will zero out those terms that describe static opinion shift $x_{k}\to x_{k}$):

$$\Pr\left[ Y_{k}\left( t+1 \right)=Y_{k}\left( t \right)+1 \right]=\sum_{s=1}^{m} \sum_{l=1}^{m} \frac{Y_{s}\left( t \right)}{N}\left( 1-\delta_{s,k} \right)\frac{Y_{l}\left( t \right)-\delta_{s,l}}{N}p_{s,l,k}.$$

Let us rewrite this equation in a more complex form that nonetheless will be useful in the following computations:

$$\Pr\left[ Y_{f}\left( t+1 \right)=Y_{f}\left( t \right)+1 \right]=\sum_{s=1}^{m} \sum_{l=1}^{m} \sum_{k=1}^{m} \frac{Y_{s}\left( t \right)}{N}\left( 1-\delta_{s,f} \right)\frac{Y_{l}\left( t \right)-\delta_{s,l}}{N}p_{s,l,k}\delta_{k,f}$$

or:

$$\Pr\left[ Y_{f}\left( t+1 \right)=Y_{f}\left( t \right)+1 \right]=\sum_{s,l,k} \frac{Y_{s}\left( t \right)}{N}\left( 1-\delta_{s,f} \right)\frac{Y_{l}\left( t \right)-\delta_{s,l}}{N}p_{s,l,k}\delta_{k,f}.$$

Analogously, the likelihood that the population of agents having opinion $x_{f}$ will decrease by one (a situation that may occur if and only if a randomly chosen agent with opinion $x_{f}$ will change this opinion) is given by:

$$\Pr\left[ Y_{f}\left( t+1 \right)=Y_{f}\left( t \right)-1 \right]=\sum_{s,l,k} \frac{Y_{s}\left( t \right)}{N}\delta_{s,f}\frac{Y_{l}\left( t \right)-\delta_{s,l}}{N}p_{s,l,k}\left( 1-\delta_{k,f} \right).$$

Given that we know agents’ opinions at time $t$, the expectation of the number of agents with opinion $x_{f}$ at the next time step $t+1$ is given by:

$$E \left[ Y_{f}\left( t+1 \right) \right]=Y_{f}\left( t \right)+\Pr\left[ Y_{f}\left( t+1 \right)=Y_{f}\left( t \right)+1 \right]-\Pr\left[ Y_{f}\left( t+1 \right)=Y_{f}\left( t \right)-1 \right].$$

Substituting expressions for $\Pr\left[ Y_{f}\left( \tau+1 \right)=Y_{f}\left( \tau\right)+1 \right]$ and $\Pr\left[ Y_{f}\left( \tau+1 \right)=Y_{f}\left( \tau\right)-1 \right]$ into the previous formula, we get:

$$E \left[ Y_{f}\left( t+1 \right) \right]=Y_{f}\left( t \right)+\sum_{s,l,k} \frac{Y_{s}\left( t \right)}{N}\frac{Y_{l}\left( t \right)-\delta_{s,l}}{N}p_{s,l,k}\left[ \left( 1-\delta_{s,f} \right)\delta_{k,f}-\delta_{s,f}\left( 1-\delta_{k,f} \right) \right]$$

or:

$$E \left[ Y_{f}\left( t+1 \right) \right]=Y_{f}\left( t \right)+\sum_{s,l,k} \frac{Y_{s}\left( t \right)}{N}\frac{Y_{l}\left( t \right)-\delta_{s,l}}{N}p_{s,l,k}\left( \delta_{k,f}-\delta_{s,f} \right).$$

Let us summarize our findings. If we know agents’ opinions at time $t$, then their opinions at time $t+1$ are given by:

$$E \left[ Y_{f}\left( t+1 \right) \right]=Y_{f}\left( t \right)+\sum_{s,l,k} \frac{Y_{s}\left( t \right)}{N}\frac{Y_{l}\left( t \right)-\delta_{s,l}}{N}p_{s,l,k}\left( \delta_{k,f}-\delta_{s,f} \right), f\in\left\{ 1,\ldots,m \right\}.$$

For large values of $N$, we can state that:

$$\frac{Y_{l}\left( t \right)-\delta_{s,l}}{N}\approx\frac{Y_{l}\left( t \right)}{N}$$

and replace $E \left[ Y_{f}\left( t+1 \right) \right]$ with $Y_{f}\left( t+1 \right)$:

$$Y_{f}\left( t+1 \right)=Y_{f}\left( t \right)+\sum_{s,l,k} \frac{Y_{s}\left( t \right)}{N}\frac{Y_{l}\left( t \right)}{N}p_{s,l,k}\left( \delta_{k,f}-\delta_{s,f} \right).$$

Establishing scaled time $\tau=\frac{t}{N}$ and scaled time step $\delta\tau=\frac{1}{N}$, we get:

$$Y_{f}\left( \tau+\delta\tau\right)=Y_{f}\left( \tau\right)+\sum_{s,l,k} \frac{Y_{s}\left( \tau\right)}{N}\frac{Y_{l}\left( \tau\right)}{N}p_{s,l,k}\left( \delta_{k,f}-\delta_{s,f} \right).$$

Dividing both sides of the equation by $N$ and introducing the normalized quantities $y_{s}\left( \tau\right)=\frac{Y_{s}\left( \tau\right)}{N}$, we obtain:

$$\frac{y_{f}\left( \tau+\delta\tau\right)-y_{f}\left( \tau\right)}{1/N}=\frac{y_{f}\left( \tau+\delta\tau\right)-y_{f}\left( \tau\right)}{\delta\tau}=\sum_{s,l,k} y_{s}\left( \tau\right)y_{l}\left( \tau\right)p_{s,l,k}\left( \delta_{k,f}-\delta_{s,f} \right)$$

Because $N$ is large, then $\delta\tau\to0$, and we finally come to the nonlinear autonomous system of differential equations:

$$\frac{dy_{f}\left( \tau\right)}{d\tau}=\sum_{s,l,k} y_{s}\left( \tau\right)y_{l}\left( \tau\right)p_{s,l,k}\left( \delta_{k,f}-\delta_{s,f} \right), f\in\left\{ 1,\ldots,m \right\} \left( A1 \right)$$

which should be equipped with the initial condition:

$$y_{f}\left( 0 \right)=y^{f}, f\in\left\{ 1,\ldots,m \right\}, \left( A2 \right)$$

where $\sum_{f=1}^{m} y^{f}=1$ and $y^{f}\in\left[ 0, 1 \right]$. Note that one of the equations in (A1) is redundant.

The equilibrium points of system (1) are given by:

$$\left\{ \begin{aligned} g_{f}=0, f\in\left\{ 1,\ldots,m \right\}, \\ y_{1}+\ldots+y_{m}=1, \\ y_{f}\in\left[ 0,1 \right], f\in\left\{ 1,\ldots,m \right\}, \end{aligned} \right.$$

where we use the notation:

$$g_{f}=\sum_{s,l,k} y_{s}y_{l}p_{s,l,k}\left( \delta_{k,f}-\delta_{s,f} \right).$$

Due to the fact that the right-hand side of (A1) is a polynomial, we can guarantee that Cauchy problem (A1)^(A2) has a unique solution, which is an analytic function of parameters $p_{s,l,k}$ and $y^{f}$.

If for some $f_{0}$ and $\tau$ one assumes that $y_{f_{0}}\left( \tau\right)=1$ (in this case, $y_{f}\left( \tau\right)=0$ for $f\neq f_{0}$), then:

$$\frac{dy_{f_{0}}\left( \tau\right)}{d\tau}=\sum_{k=1}^{m} \left( y_{f_{0}}\left( \tau\right) \right)^{2}p_{f_{0},f_{0},k}\left( \delta_{k,f_{0}}-1 \right)=-\sum_{k\in\left\{ 1,\ldots,m \right\}/f_{o}} \left( y_{f_{0}}\left( \tau\right) \right)^{2}p_{f_{0},f_{0},k}\leq0.$$

Hence, the mean-field approximation does not violate the definition of quantities $y_{f}$: the fraction of individuals espousing a particular opinion cannot go beyond interval $\left[ 0,1 \right]$.

**Appendix 4. System (A1) (system (1) in Main Manuscript) in the case of the binary opinion space**

If $m=2$, which corresponds to the binary opinion space, then system (A1) takes the relatively simple form:

$$\left\{ \begin{aligned} \dot{y}_{1}=-p_{1,1,2}y_{1}^{2}+\left( p_{2,1,1}-p_{1,2,2} \right)y_{1}y_{2}+p_{2,2,1}y_{2}^{2}, \\ \dot{y}_{2}=p_{1,1,2}y_{1}^{2}+\left( p_{1,2,2}-p_{2,1,1} \right)y_{1}y_{2}-p_{2,2,1}y_{2}^{2}. \end{aligned} (A3) \right.$$

Note that one of two equations in system (A3) is unnecessary. Substituting $y_{1}=1-y_{2}$ into the first one, we obtain:

$$g_{1}=ay_{1}^{2}+by_{1}+c,$$

where:

$$\begin{matrix} a=-p_{1,1,2}+p_{1,2,2}-p_{2,1,1}+p_{2,2,1}, \\ b=p_{2,1,1}-p_{1,2,2}-2p_{2,2,1}, \\ c=p_{2,2,1}. \end{matrix}$$

If $y_{1}^{*}\in\left[ 0,1 \right]$ is an equilibrium point, then it should admit equation:

$$g_{1}\left( y_{1}^{*} \right)=0.$$

The sign of the quantity:

$$\dot{g}_{1}\left( y_{1}^{*} \right)=2ay_{1}^{*}+b$$

determines this equilibrium's stability properties.

**Appendix 5. Dataset organization**

Dataset contains information on the dynamics of political preferences of a large-scale sample $I$ (approximately 1.6 M) of VKontakte (the most popular Russian social network) users. The sample was made by randomly choosing active (at least one platform interaction per month) individuals whose account is available (not banned, deleted, or hidden by privacy settings), this one older than 17 years, is credited as Russian, and who follow no less than 10 (to ensure that only sufficiently active information consumers are under consideration) and no more than 200 (to avoid accounts that may pursue goals different from simple information consumption) information sources^6,7^. Note that these numbers appear not only from simple intuition: preliminary experiments revealed that opinion estimations are more precise when such individuals are excluded from the test sample^11^. Further, the sample was additionally cleared of isolated subgroups of online friends in such a manner that the resulting social network (whereby edges represent online friends) consists of one (giant) connected component. Note that only 0.7% of all nodes were removed during this procedure.

Users’ opinions were estimated on a continuous opinion scale $\left[ 0,1 \right]$, where extreme positions 0 and 1 represent maximal opposition and support for the current Russian government correspondingly. Dataset is organized as three opinion snapshots made in February, July, and December 2018: $\hat{o}\left( t_{1} \right),\hat{o}\left( t_{2} \right),$ and $\hat{o}\left( t_{3} \right)$ correspondingly, where the symbol “^” emphasizes that it provides only estimations of opinions. These estimations were made by applying a trained logit model. Users’ subscriptions to VKontakte information sources were encoded using the one-hot encoding strategy and were employed as features, reflecting the fact that individuals tend to consume information coherent with their current opinions. As such, the dynamics of users’ subscriptions to information sources should reflect the dynamics of their opinions.

The second component of Dataset is the symmetric adjacency matrix $\hat{A}$, the elements $\hat{a}_{ij}\in\left\{ 0,1 \right\}$ of which represent friendship connections between the sample users at time point $t_{2}$. As aforementioned, the corresponding social network is a connected graph.

As a result, each user $i$ from Dataset is characterized by the sequence of their own opinions $\hat{o}_{i}\left( t_{1} \right),\hat{o}_{i}\left( t_{2} \right),$ and $\hat{o}_{i}\left( t_{3} \right)$ and by the sequence of average opinions of their friends $\hat{o}_{-i}\left( t_{1} \right),\hat{o}_{-i}\left( t_{2} \right),$ and $\hat{o}_{-i}\left( t_{3} \right)$, where

$$\hat{o}_{-i}\left( t \right)=\frac{\sum_{j} \hat{a}_{ij}o_{j}\left( t \right)}{\sum_{j} \hat{a}_{ij}}$$

Note that Dataset is built under the assumption that friendship connections are static, representing one of its main disadvantages.

**Appendix 6. Estimating transition matrices**

Let us now describe how we integrate Dataset information into the model. We will consider the case $m=2$; other situations are elaborated analogously. We discretize the empirical opinion scale $\left[ 0,1 \right]$ by endowing users who have opinion values from the interval $\left[ 0,0.5 \right]$ with new opinions $x_{1}$ (say, $x_{1}=-1$). Analogously, those who have opinions from the interval $\left[ 0.5,1 \right]$ are marked with $x_{2}$ (say, $x_{2}=1$). A similar procedure is applied on average opinions of users’ friends. Next, for each $s,l$, and $k$, we calculate quantity $p_{s,l,k}$ as follows:

$$p_{s,l,k}=\frac{\#\left\{ i\in I | \left( \hat{o}_{i}\left( t_{1} \right)=x_{s} \right)\&\left( \hat{o}_{-i}\left( t_{1} \right)=x_{l} \right)\&\left( \hat{o}_{i}\left( t_{2} \right)=x_{k} \right) \right\}}{\#\left\{ i\in I | \left( \hat{o}_{i}\left( t_{1} \right)=x_{s} \right)\&\left( \hat{o}_{-i}\left( t_{1} \right)=x_{l} \right) \right\}}, (A4)$$

where $\#\left\{ \ldots\right\}$ denotes the cardinal number of the set. To put it simply, (A4) is the fraction of individuals who made opinion change $x_{s}\to x_{k}$ among those whose opinion is $x_{s}$ and whose friends’ average opinion is equal to $x_{l}$. To avoid noise, we additionally require that the opinion shift should have a magnitude of more than 0.05 on the continuous scale. Note that in (A4), we first use two snapshots to calibrate the transition matrix’s elements. Acting analogously, one can estimate the transition matrix from second and third opinion snapshots:

$$p_{s,l,k}=\frac{\#\left\{ i\in I | \left( \hat{o}_{i}\left( t_{2} \right)=x_{s} \right)\&\left( \hat{o}_{-i}\left( t_{2} \right)=x_{l} \right)\&\left( \hat{o}_{i}\left( t_{3} \right)=x_{k} \right) \right\}}{\#\left\{ i\in I | \left( \hat{o}_{i}\left( t_{2} \right)=x_{s} \right)\&\left( \hat{o}_{-i}\left( t_{2} \right)=x_{l} \right) \right\}}$$

We should say that substantial opinion diversity, presented in Dataset, ensures that all combinations of $x_{s}$ and $x_{l}$ are available in sufficient quantity, providing the opportunity to estimate all the transition matrix elements (see Table A1).

Table A1

Joint distribution of users’ opinions and opinions of their online friends (first opinion snapshot, $m=10$)

|  | | Average opinion of online friends | | | | | | | | | |
| --- | --- | --- | --- | --- | --- | --- | --- | --- | --- | --- | --- |
|  |  | $x_{1}$ | $x_{2}$ | $x_{3}$ | $x_{4}$ | $x_{5}$ | $x_{6}$ | $x_{7}$ | $x_{8}$ | $x_{9}$ | $x_{10}$ |
| User’s opinion | $x_{1}$ | 371 | 699 | 3,833 | 21,084 | 24,955 | 6,934 | 1,332 | 376 | 147 | 44 |
|  | $x_{2}$ | 246 | 486 | 2,761 | 19,626 | 27,570 | 7,475 | 1,359 | 409 | 124 | 45 |
|  | $x_{3}$ | 323 | 595 | 3,307 | 27,307 | 49,928 | 15,408 | 2,750 | 745 | 217 | 75.0 |
|  | $x_{4}$ | 624 | 1,002 | 5,064 | 45,533 | 105,023 | 36,341 | 6,148 | 1,546 | 544 | 151 |
|  | $x_{5}$ | 1,318 | 2,111 | 9,915 | 90,305 | 277,315 | 107,562 | 18,666 | 4,586 | 1,575 | 405 |
|  | $x_{6}$ | 825 | 1,253 | 4,932 | 41,661 | 193,936 | 100,509 | 20,220 | 5,237 | 1,833 | 439 |
|  | $x_{7}$ | 470 | 614 | 2,142 | 14,414 | 78,395 | 56,749 | 14,657 | 4,079 | 1,293 | 350 |
|  | $x_{8}$ | 245 | 336 | 1,206 | 6,922 | 37,826 | 34,854 | 11,471 | 3,495 | 1,170 | 319 |
|  | $x_{9}$ | 179 | 222 | 722 | 3,451 | 17,376 | 19,377 | 8,136 | 2,703 | 1,006 | 273 |
|  | $x_{10}$ | 58 | 63 | 254 | 1,305 | 5,553 | 5,716 | 2,679 | 1,063 | 457 | 119 |

**Appendix 7. Stability analysis in the case** $\boldsymbol{m=3}$**.**


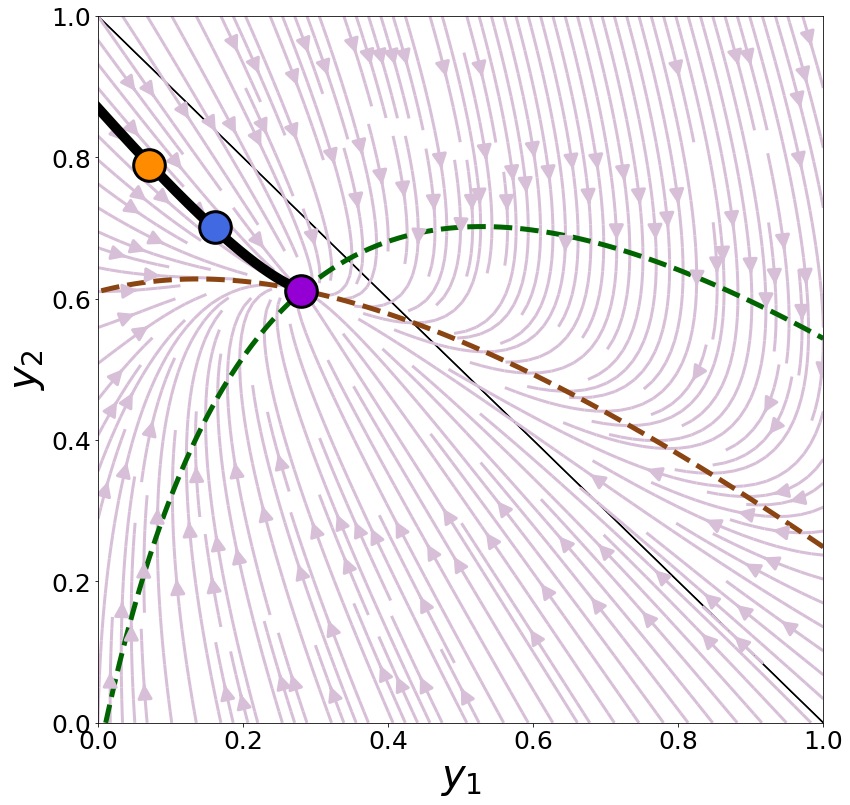


Figure A1. Dashed lines portray the curves $g_{1}\left( y_{1},y_{2} \right)=0$ (green) and $g_{2}\left( y_{1},y_{2} \right)=0$ (brown). Their intersection marks the equilibrium point (the violet circle) and the straight black solid line plots $y_{1}+y_{2}=1$. We are interested in the area $y_{1}+y_{2}\leq1$ beneath this line. The phase portrait of the system demonstrates that the equilibrium point is an asymptotically stable nodal sink (the Jacobian matrix at the equilibrium point has two different negative eigenvalues), which attracts the system regardless of its initial point. The blue and orange circles lying on the same phase (bold black) curve represent opinion distributions $y_{1}=0.161,y_{2}=0.702,y_{3}=0.137$ (see Table 1 from Main Manuscript) and $y_{1}=0.08,y_{2}=0.78,y_{3}=0.14$ (see (6) in Main Manuscript) correspondingly.

**Appendix 8. Definition of the assortativity coefficient**

The assortativity coefficient

$$C\left( A,o\left( t \right) \right)=\frac{\sum_{i,j} \left( a_{ij}-\frac{k_{i}k_{j}}{2q} \right)o_{i}\left( t \right)o_{j}\left( t \right)}{\sum_{i,j} \left( k_{i}\delta_{ij}-\frac{k_{i}k_{j}}{2q} \right)o_{i}\left( t \right)o_{j}\left( t \right)}\in\left[ -1,1 \right] (A5)$$

measures whether the system at hand is homophilic (connected nodes tend to have similar opinions). In (A5), vector $o\left( t \right)=\left[ \begin{matrix} o_{1}\left( t \right) & \ldots& o_{N}\left( t \right) \end{matrix} \right]^{T}$ stands for current agents’ opinions, adjacency matrix $A=\left[ a_{ij} \right]\in\left\{ 0,1 \right\}^{N\times N}$ describes the structure of social network $G$, and $q$ is the number of edges in the network. $k_{i}$ represents node $i$’s degree: $k_{i}=\sum_{i=1}^{N} a_{ij}$. Put simply, (A5) measures how similar the neighboring opinions are, compared with the configuration in which edges are placed at random. For homophilic networks (most empirically observed social networks are homophilic), metric (A5) takes positive values (assortative mixing). To compute the assortativity coefficient, we use $x_{1}=0,x_{2}=1,\ldots,x_{m}=m-1$. Instead, if we calculate the dissimilarity coefficient, then we reinitialize opinion values to make them lie in the interval $\left[ -1, 1 \right]$: $x_{1}=-1,\ldots,x_{m}=1$.

**Appendix 9. Information on synthetic networks used in simulation experiments**

Table A2

Properties of synthetic networks

| Network model | Parameter(s) | Brief description |
| --- | --- | --- |
| Erdős–Rényi | 15000 edges | The model places a predefined number of edges between the nodes at random. The resulting network has no common features with real social graphs (ignoring sparsity that can be obtained for small values of the parameter) but may serve as their simplest (apart from the complete graph) approximation. |
| Random geometric graph | Threshold distance 0.05 | The nodes are randomly placed in the unit square. Each pair of nodes is connected if and only if they are distant for no more than the threshold value. The model allows us to obtain the “structured” graphs containing communities (organized geographically), the presence of which is a prominent signature of real social networks. |
| Watts–Strogatz | Each node is connected to 15 nearest ones, probability of rewiring $p_{rew}=0$ (WS1) | Initially, nodes are placed in the ring topology, in which each node is connected to a predefined number of nearest ones. Edges are rewired at random with the probability $p_{rew}$. If $p_{rew}=0$, then we obtain clustered networks (with an average clustering coefficient $\approx0.69$) that feature high values of average path length  ($\approx72$). A tiny increase in the value of the rewiring probability ($p_{rew}=0.01$) leads to networks that are still highly clustered (average clustering $\approx0.67$) but characterized by a small average path length ($\approx7.6$)—so-called small-world networks. If $p_{rew}=1$, then the model generates graphs with no clustering but with even lower values of the average path length ($\approx3$). |
|  | Each node is connected to 15 nearest ones, $p_{rew}=0.01$ (WS2) |  |
|  | Each node is connected to 15 nearest ones, $p_{rew}=1$ (WS3) |  |
| Barabási–Albert | Each new node is attached to seven already existing ones | The model adds new nodes in the system sequentially, connecting them with existing ones at random, following the preferential attachment rule. The resulting networks follow the power-law degree distribution, which is widely observed in real social networks. |

Note: all model parameters are tuned to ensure that the resulting networks are connected and have approximately the same density (or the same number of edges $q$).

**Appendix 10. Organization of the estimated transition matrix in the case** $\boldsymbol{m=10}$

Applying the algorithm presented in Appendix 6, one can estimate the transition matrix in the case of the tenfold opinion space. In tables A3–A5, we present some slices of this matrix (rounded to 3 decimal places). In these tables, bolded columns indicate the probabilities of holding the current opinion (the most popular strategy observed). Importantly, the presence of both positive and negative influence can be found in these slices: if we increase $\left| x_{s}-x_{l} \right|$, then the probability that a user’s opinion will be changed is raised, and changes both towards and outwards (see Table A4) influencing opinion become more likely. A more profound analysis of Dataset opinion dynamics can be found in Ref.^7^. The full version of the tenfold transition matrix can be found in the Online Supplementary Materials.

Table A3

Slice $P_{1,:,:}$

| **0.942** | 0.038 | 0.003 | 0.009 | 0.009 | 0.000 | 0.000 | 0.000 | 0.000 | 0.0 |
| --- | --- | --- | --- | --- | --- | --- | --- | --- | --- |
| **0.938** | 0.044 | 0.005 | 0.006 | 0.005 | 0.000 | 0.003 | 0.000 | 0.000 | 0.0 |
| **0.945** | 0.042 | 0.007 | 0.003 | 0.002 | 0.000 | 0.000 | 0.000 | 0.000 | 0.0 |
| **0.947** | 0.037 | 0.008 | 0.004 | 0.003 | 0.001 | 0.000 | 0.000 | 0.000 | 0.0 |
| **0.939** | 0.036 | 0.009 | 0.006 | 0.005 | 0.003 | 0.001 | 0.001 | 0.000 | 0.0 |
| **0.924** | 0.043 | 0.009 | 0.008 | 0.007 | 0.004 | 0.002 | 0.001 | 0.001 | 0.0 |
| **0.925** | 0.045 | 0.009 | 0.006 | 0.008 | 0.002 | 0.002 | 0.002 | 0.000 | 0.0 |
| **0.908** | 0.065 | 0.006 | 0.003 | 0.000 | 0.009 | 0.003 | 0.006 | 0.000 | 0.0 |
| **0.896** | 0.067 | 0.037 | 0.000 | 0.000 | 0.000 | 0.000 | 0.000 | 0.000 | 0.0 |
| **0.881** | 0.048 | 0.000 | 0.000 | 0.000 | 0.000 | 0.048 | 0.024 | 0.000 | 0.0 |

Table A4

Slice $P_{5,:,:}$

| 0.002 | 0.001 | 0.011 | 0.080 | **0.854** | 0.051 | 0.002 | 0.000 | 0.000 | 0.000 |
| --- | --- | --- | --- | --- | --- | --- | --- | --- | --- |
| 0.001 | 0.004 | 0.009 | 0.067 | **0.873** | 0.040 | 0.004 | 0.001 | 0.001 | 0.000 |
| 0.001 | 0.004 | 0.010 | 0.070 | **0.874** | 0.038 | 0.003 | 0.000 | 0.000 | 0.000 |
| 0.001 | 0.002 | 0.008 | 0.060 | **0.896** | 0.031 | 0.001 | 0.000 | 0.000 | 0.000 |
| 0.001 | 0.001 | 0.005 | 0.054 | **0.895** | 0.041 | 0.002 | 0.001 | 0.000 | 0.000 |
| 0.000 | 0.001 | 0.005 | 0.059 | **0.871** | 0.059 | 0.004 | 0.000 | 0.000 | 0.000 |
| 0.001 | 0.001 | 0.005 | 0.068 | **0.837** | 0.078 | 0.008 | 0.001 | 0.000 | 0.000 |
| 0.000 | 0.002 | 0.008 | 0.068 | **0.827** | 0.083 | 0.011 | 0.003 | 0.000 | 0.000 |
| 0.000 | 0.001 | 0.005 | 0.066 | **0.826** | 0.090 | 0.009 | 0.003 | 0.000 | 0.001 |
| 0.000 | 0.003 | 0.013 | 0.090 | **0.790** | 0.092 | 0.005 | 0.005 | 0.000 | 0.003 |

Note: probabilities of negative shifts are denoted with lower font size numbers

Table A5

Slice $P_{10,:,:}$

| 0.000 | 0.0 | 0.000 | 0.000 | 0.000 | 0.000 | 0.018 | 0.000 | 0.125 | **0.857** |
| --- | --- | --- | --- | --- | --- | --- | --- | --- | --- |
| 0.000 | 0.0 | 0.000 | 0.000 | 0.000 | 0.018 | 0.018 | 0.000 | 0.161 | **0.804** |
| 0.000 | 0.0 | 0.005 | 0.000 | 0.005 | 0.005 | 0.000 | 0.014 | 0.096 | **0.876** |
| 0.001 | 0.0 | 0.000 | 0.000 | 0.006 | 0.005 | 0.005 | 0.005 | 0.081 | **0.897** |
| 0.000 | 0.0 | 0.000 | 0.001 | 0.002 | 0.004 | 0.004 | 0.005 | 0.064 | **0.919** |
| 0.001 | 0.0 | 0.000 | 0.001 | 0.001 | 0.002 | 0.005 | 0.005 | 0.076 | **0.909** |
| 0.000 | 0.0 | 0.000 | 0.000 | 0.002 | 0.002 | 0.006 | 0.007 | 0.079 | **0.904** |
| 0.001 | 0.0 | 0.001 | 0.000 | 0.000 | 0.003 | 0.006 | 0.005 | 0.074 | **0.910** |
| 0.000 | 0.0 | 0.000 | 0.000 | 0.005 | 0.000 | 0.002 | 0.014 | 0.079 | **0.900** |
| 0.000 | 0.0 | 0.000 | 0.000 | 0.000 | 0.000 | 0.000 | 0.018 | 0.071 | **0.912** |

**Appendix 11. Robustness against initial opinions**

**
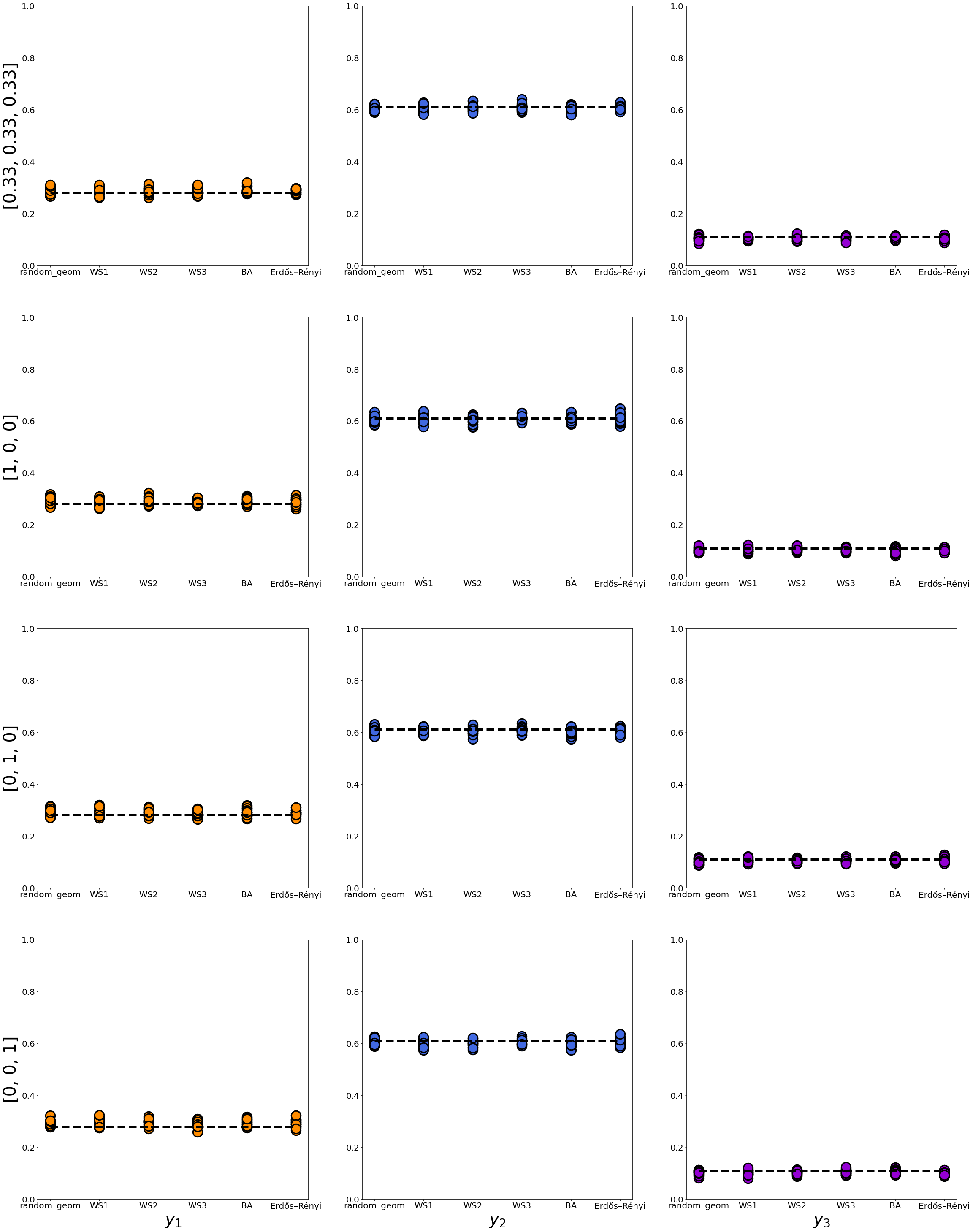
**

Figure A2. The panels plot how the limiting values of public opinion variables vary with configurations of initial opinions.

**Appendix 12. Two-matrix simulation run for Model 1**

In this Appendix, we demonstrate that within the baseline model, we cannot reach the desired level of homophily unless the system is already homophilic prior to the beginning of a simulation run. One can make the system homophilic before an experiment by, for example, employing one more (auxiliary) transition matrix that has to prepare the (initially random) system by making it sufficiently homophilic. For example, the following transition matrix, inspired by the Voter model, can achieve this purpose:

$$P_{1,:,:}=P_{2,:,:}=P_{3,:,:}=\left[ \begin{matrix} 1 & 0 & 0 \\ 0 & 1 & 0 \\ 0 & 0 & 1 \end{matrix} \right]. (A6)$$

In this case, however, the assortativity coefficient will decrease until it reaches the asymptotic value (see Figure A2), and at the reference point, one will observe a decreasing trend. These dynamical patterns stand in contradiction with what we observe in empirics.


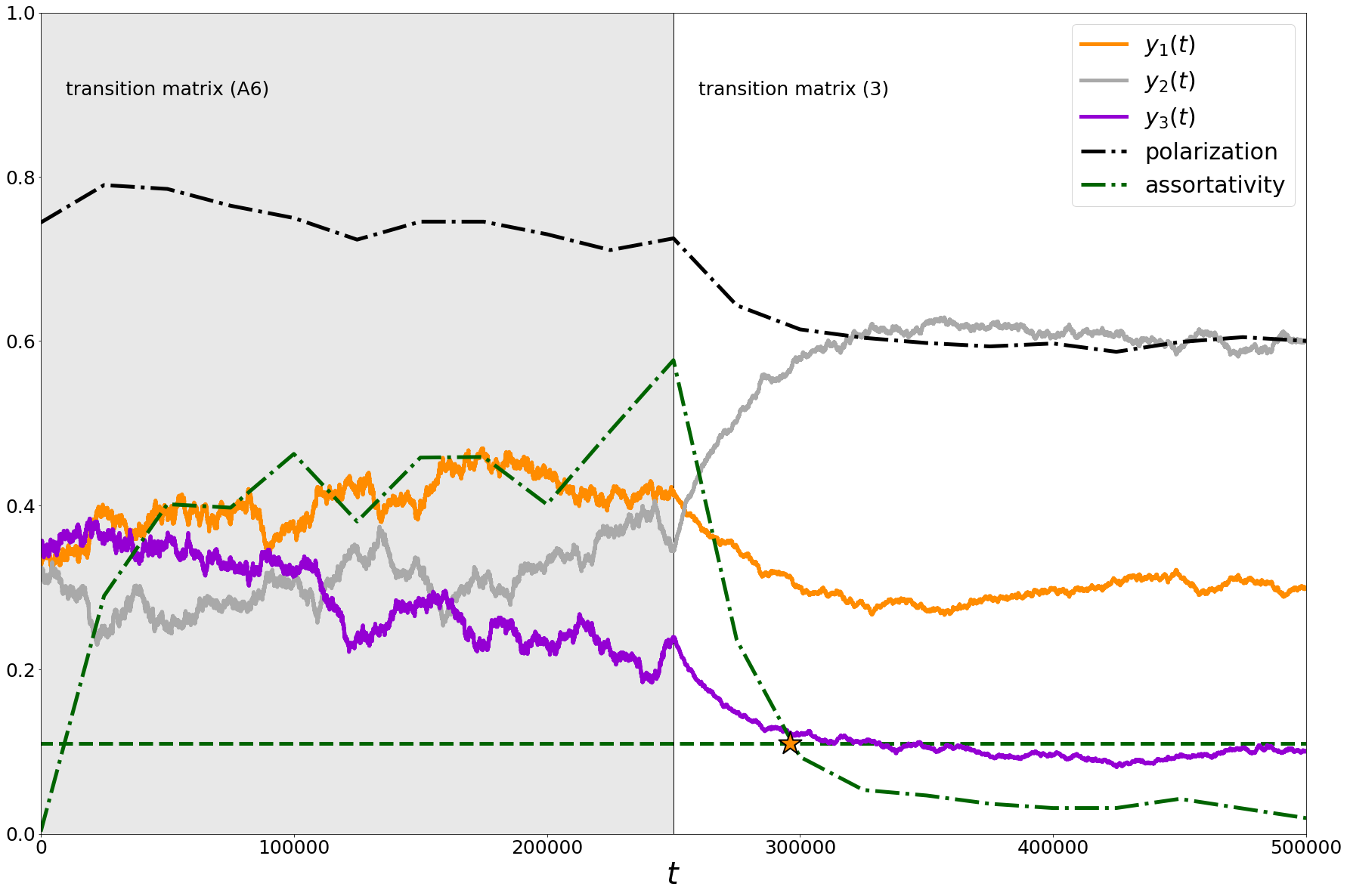


Figure A3. Simulation run demonstrating how two different transition matrices can govern opinion dynamics in a sequential fashion. Dashed horizontal line represents the reference value of the assortativity coefficient from Table 1 (we use the first opinion snapshot). As long as $t<250,000$, transition matrix (A5) is in charge (see gray area), and the assortativity coefficient reaches sufficiently high values. At time $t=250,000$ (the edge between gray and white areas), the system switches to transition matrix (3). Because the model is memoryless, the macroscopic metrics quickly converge to the asymptotic values prescribed by transition matrix (3), leaving behind transition matrix (A6). At time moment $t\approx300,000$, the assortativity coefficient coincides with the reference value (the intersection is indicated by the orange star-like marker). However, around this point, the assortativity curve demonstrates a decrease, behavior that disagrees with the empirical trend.

**Appendix 13. Effect of errors in estimated transition matrices on limiting values of public opinion variables.**


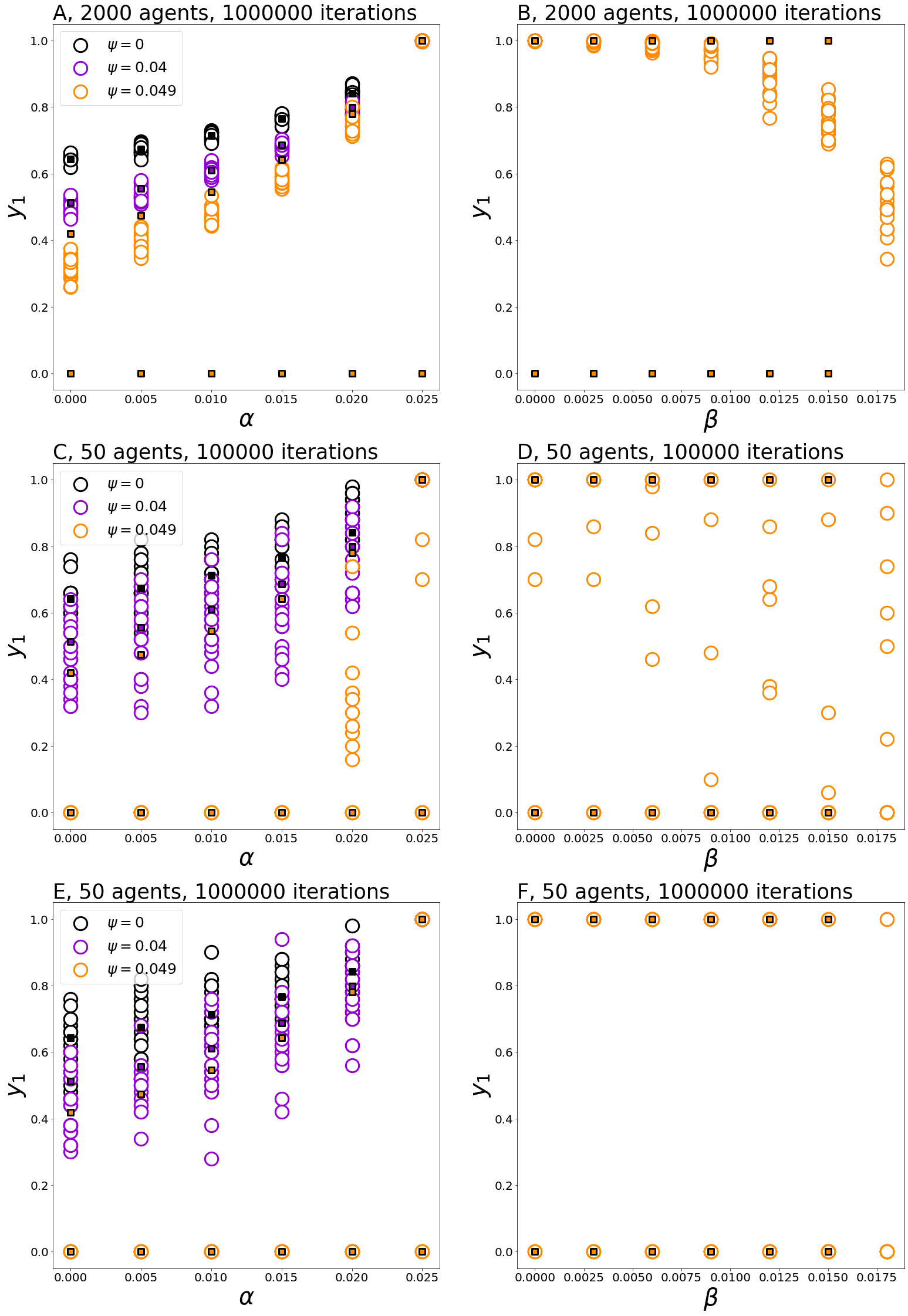


Figure A4. The panels compare mean-field predictions of the model behavior (colored squares) against simulations (white circles) in Scenarios 1 (panels A, C, E) and 2 (panels B, D, F). Panels A and B represent experiments with 2000 agents whose opinions were initialized from the uniform distribution. In panels C, D, E, and F, simulations included 50 agents and were started from distribution $y_{1}=0.1,y_{2}=0.9$. Presented simulations were conducted on random geometric networks.

Figures A5–A9 below compare mean-field predictions of the model behavior (colored squares) against simulations (white circles) in Scenarios 1 (panels A) and 2 (panels B) across different network topologies (A5 – WS1 networks, A6 – Erdős–Rényi networks, A7 – BA networks, A8 – WS3 networks, A9 – complete networks). The presented results were obtained in experiments with 2000 agents whose opinions were initialized from the uniform distribution. Limiting opinion distributions were obtained after 1,000,000 iterations.

*
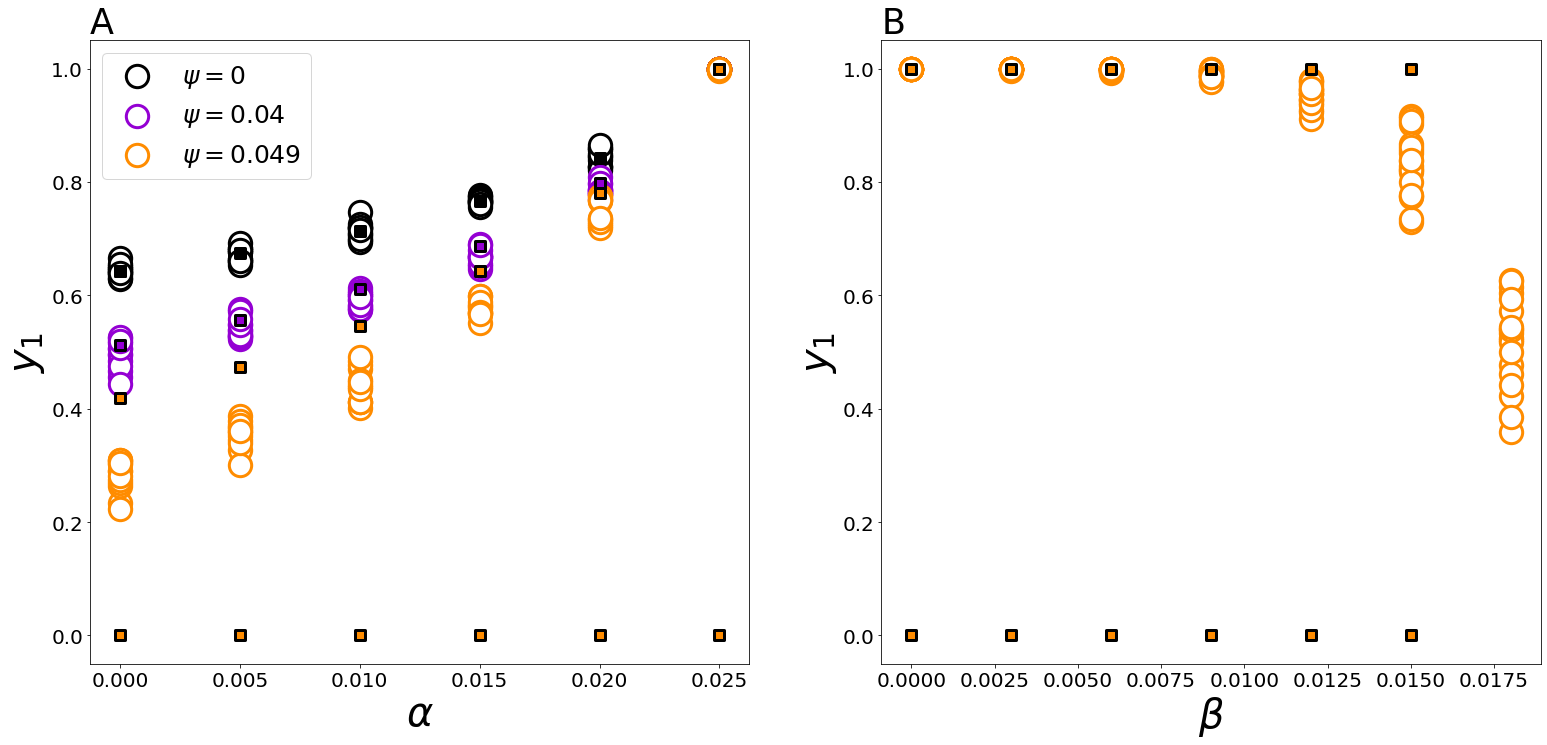
*

Figure A5.


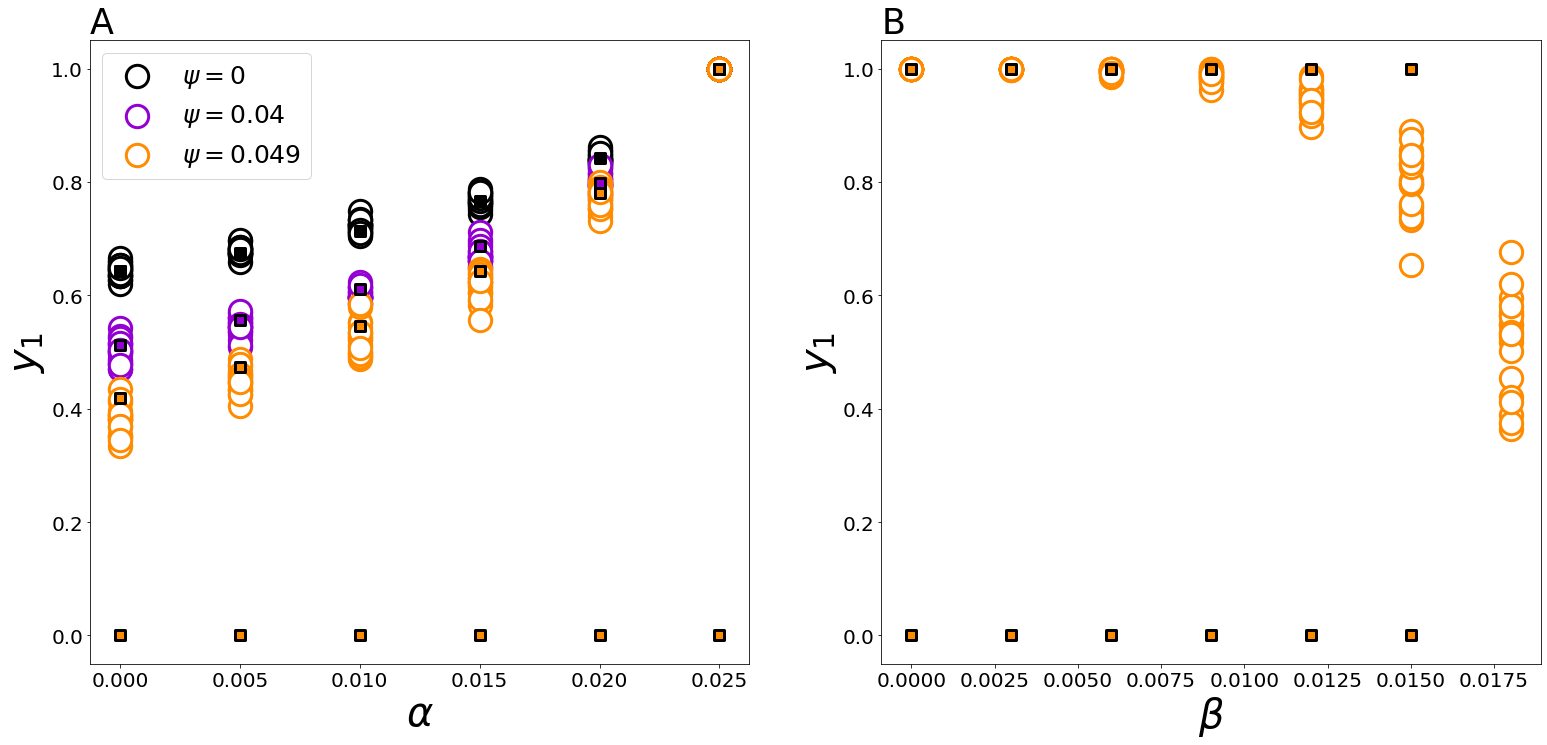


Figure A6.


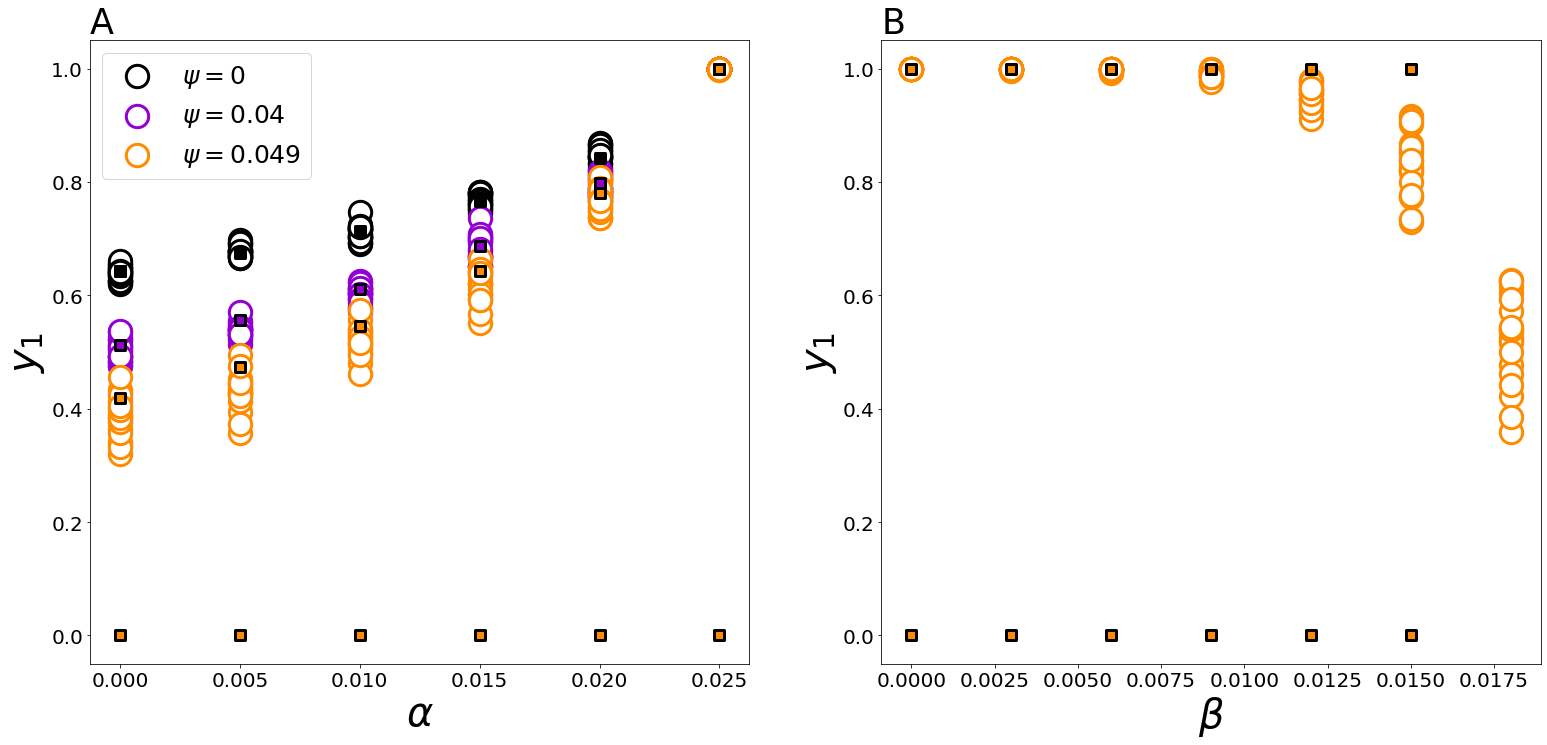


Figure A7.


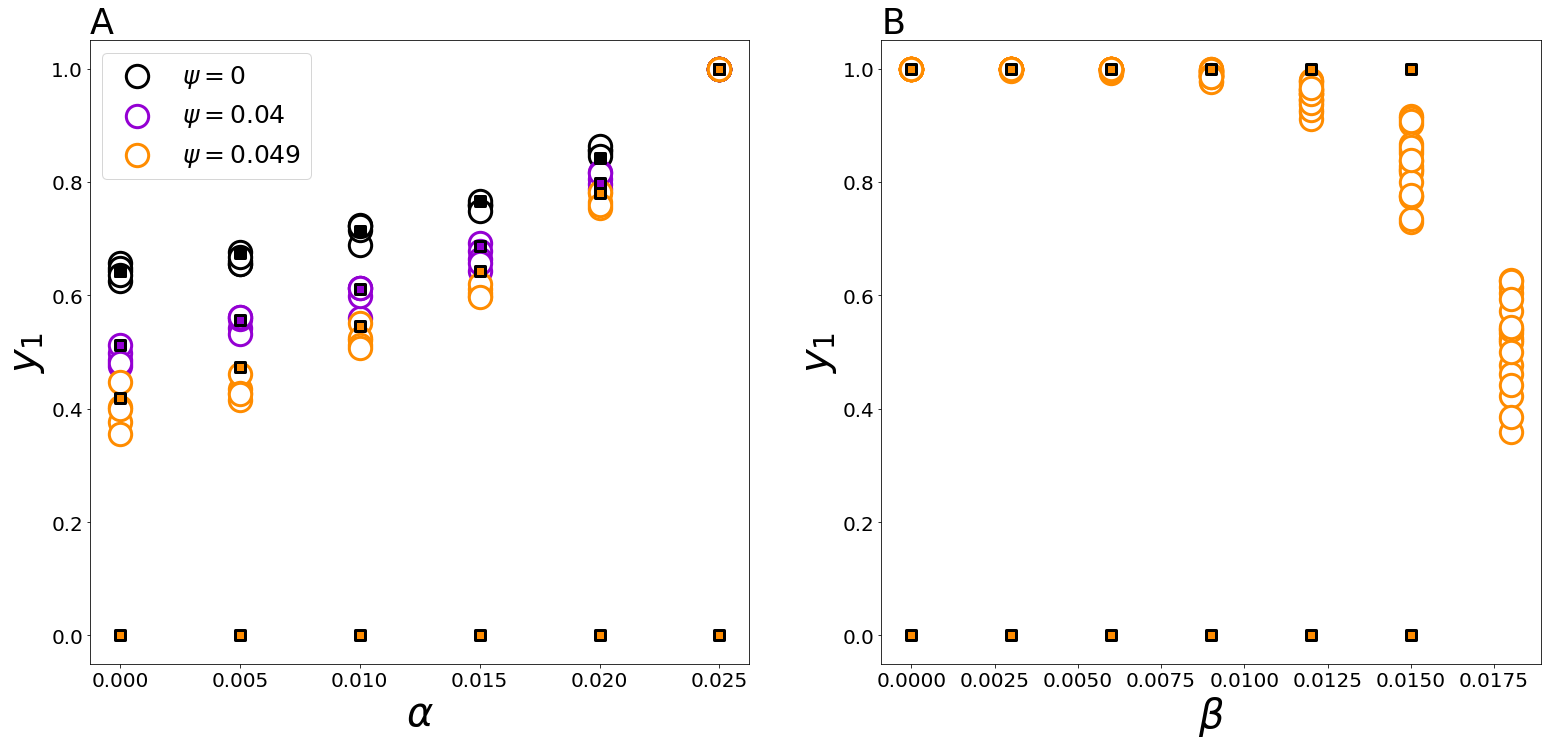


Figure A8.


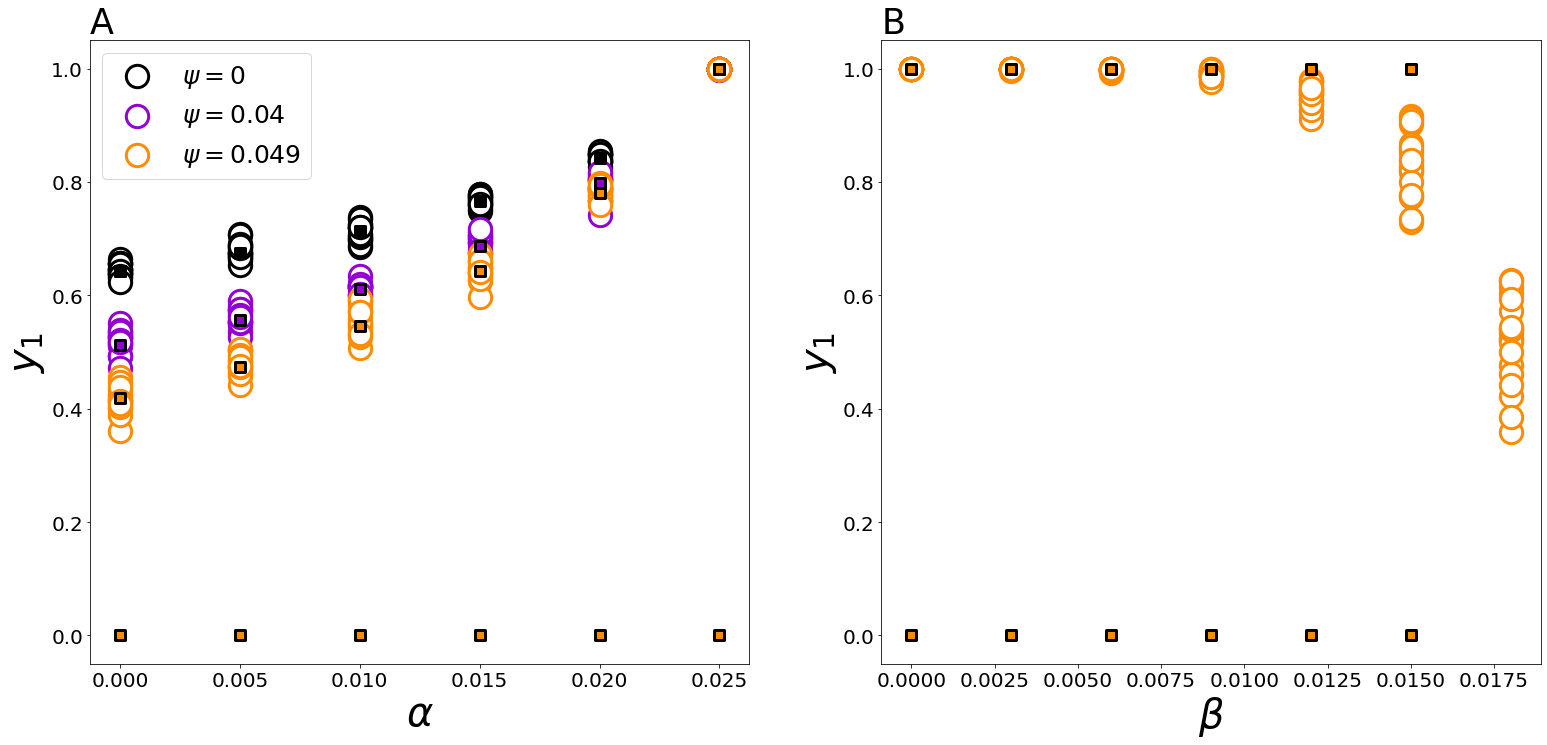


Figure A9.

**Appendix 13. Effect of errors in estimated transition matrices on assortativity coefficient.**


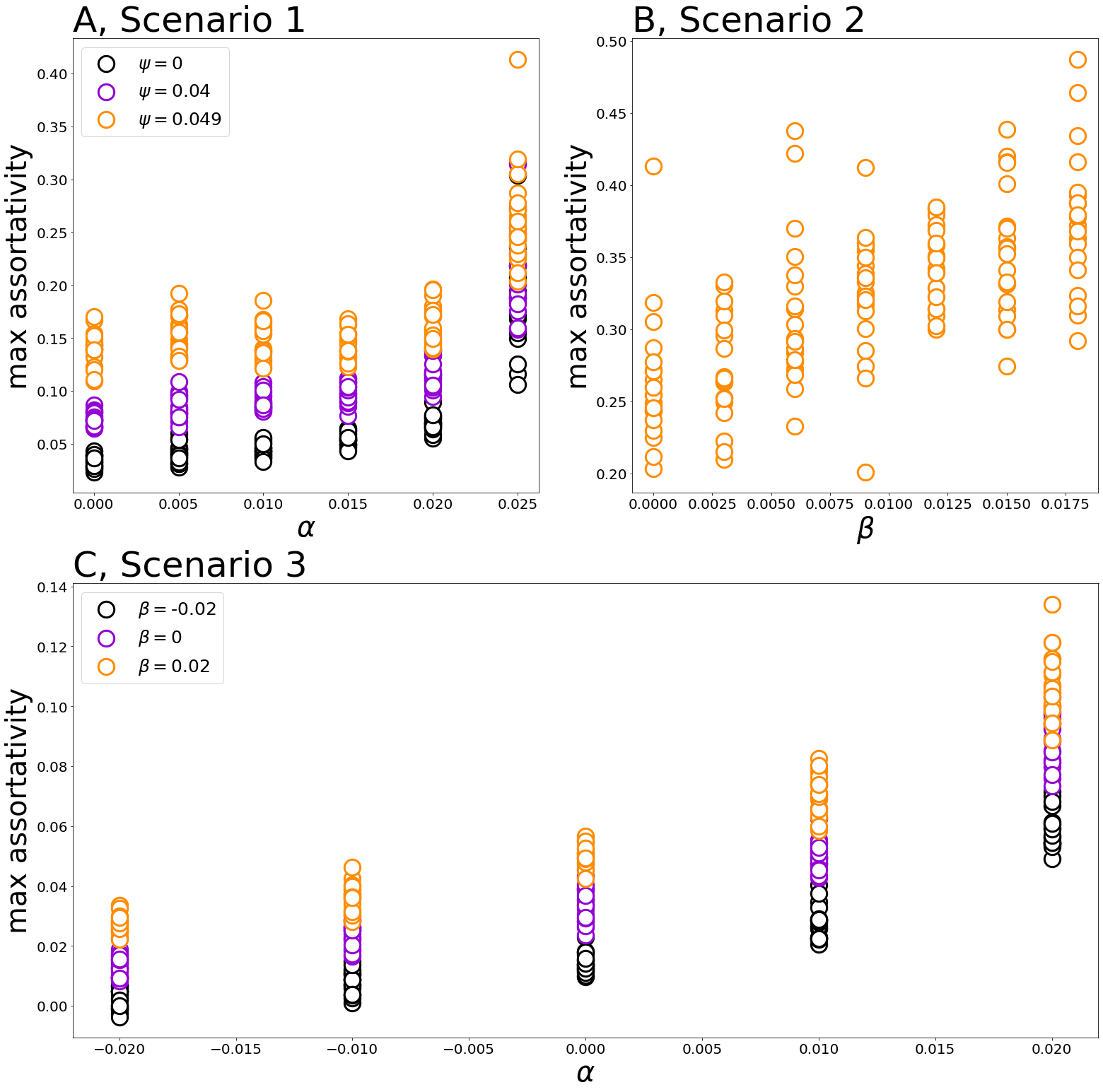


Figure A10*.* Maximal value of assortativity (during a single experiment) as a function of errors in the estimated transition matrix across different Scenarios. Presented results were obtained on random geometric networks.


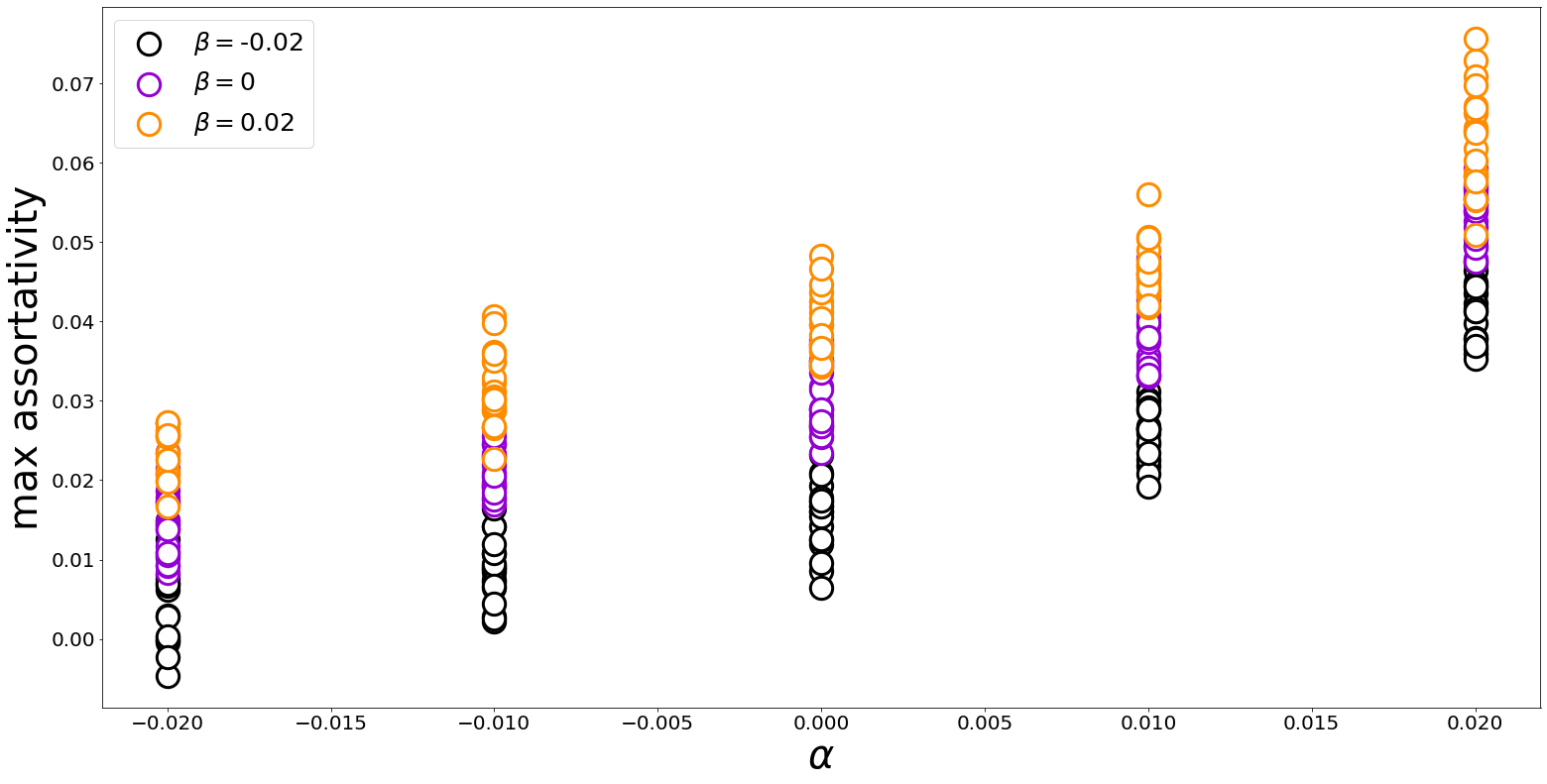


Figure A11. Maximal value of assortativity (during a single experiment) as a function of errors in the estimated transition matrix (Scenario 3). Presented results were obtained on Erdős–Rényi networks.

**Appendix 14. Effect of topology on assortativity coefficient for different values of selectivity and personalization**


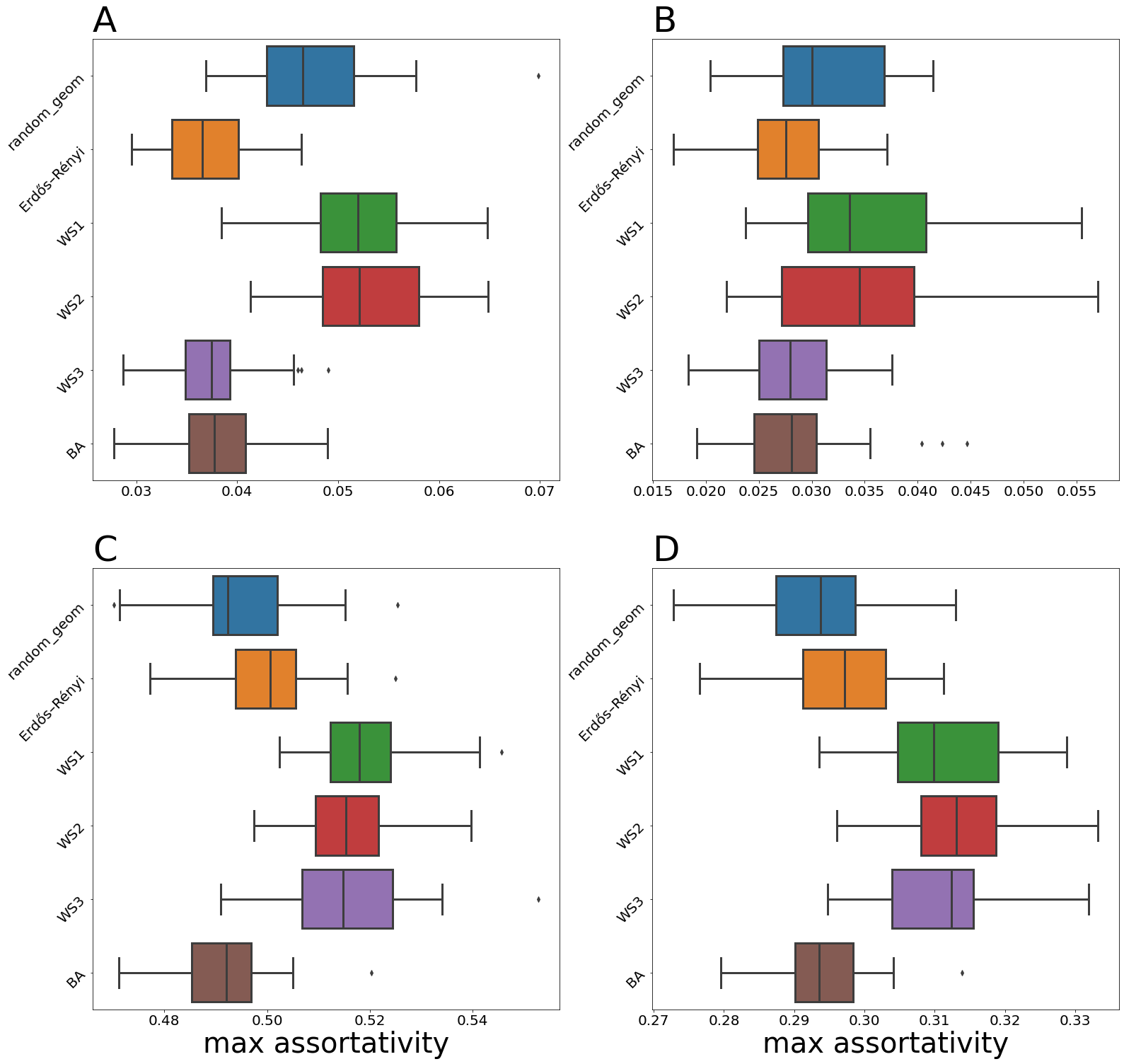


Figure A12. Effect of topology on assortativity coefficient for different combinations of selectivity and personalization levels: $\gamma=0,\delta=0$ (panel A), $\gamma=0,\delta=0.4$ (panel B), $\gamma=0.6,\delta=0$ (panel C), $\gamma=1,\delta=0.8$ (panel D). All results are derived for $m=10$.

**Appendix 15. Estimated transition matrix calculated for second and third snapshots**

Using the second and third opinion snapshots from Dataset, one can obtain the following transition matrix (rounded to 3 decimal places):

$$P_{1,:,:}=\left[ \begin{matrix} 0.976 & 0.024 & 0 \\ 0.967 & 0.033 & 0 \\ 0.94 & 0.058 & 0.002 \end{matrix} \right],P_{2,:,:}=\left[ \begin{matrix} 0.027 & 0.966 & 0.006 \\ 0.016 & 0.977 & 0.008 \\ 0.014 & 0.96 & 0.026 \end{matrix} \right],P_{3,:,:}=\left[ \begin{matrix} 0.002 & 0.065 & 0.933 \\ 0.001 & 0.048 & 0.951 \\ 0 & 0.036 & 0.963 \end{matrix} \right]. (A7)$$

This transition matrix (if $\gamma=0$ and $\delta=0$) marks the equilibrium point $y_{1}^{*}\approx0.34,y_{2}^{*}\approx0.56,y_{3}^{*}\approx0.1$ (see Figure A13). However, after implementing $\gamma^{*}=0.13$ and $\delta^{*}=0.15$, we obtain that the system ends up in the opinion distribution $y_{1}^{*}\approx0.331,y_{2}^{*}\approx0.546,y_{3}^{*}\approx0.123$ (see Table A6).


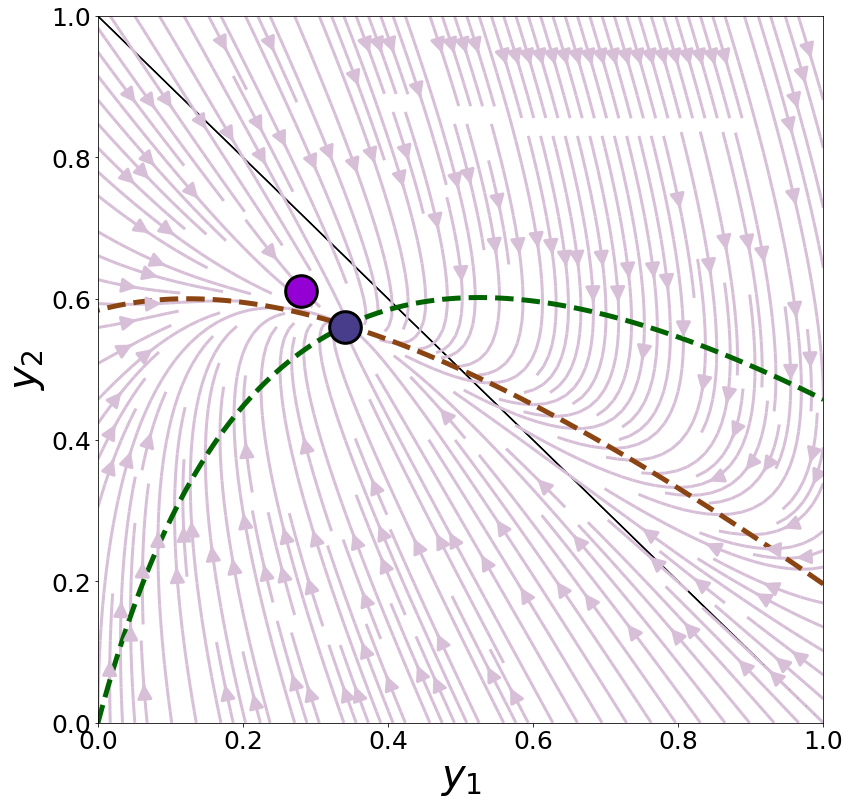


Figure A13. Phase portrait for transition matrix (A7). The dark blue circle plots the equilibrium point $y_{1}^{*}\approx0.34,y_{2}^{*}\approx0.56,y_{3}^{*}\approx0.1$. The violet circle marks the equilibrium point for transition matrix (3) from Main Manuscript.

Table A4

Equilibrium points obtained for transition matrices (3) and (A7) in Models 1 and 2

| Transition matrix | Selectivity | Personalization | Equilibrium point | | |
| --- | --- | --- | --- | --- | --- |
|  |  |  | $y_{1}$ | $y_{2}$ | $y_{3}$ |
| (3) from Main Manuscript | 0 | 0 | 0.28 | 0.611 | 0.109 |
| (A7) | 0 | 0 | 0.34 | 0.56 | 0.1 |
| (3) from Main Manuscript | 0.13 | 0.15 | 0.299 | 0.591 | 0.111 |
| (A7) | 0.13 | 0.15 | 0.331 | 0.546 | 0.123 |

**References**

1. Clifford, P. & Sudbury, A. A model for spatial conflict. *Biometrika* **60**, 581–588 (1973).

2. Flache, A. *et al.* Models of Social Influence: Towards the Next Frontiers. *J. Artif. Soc. Soc. Simul.* **20**, (2017).

3. Kurahashi-Nakamura, T., Mäs, M. & Lorenz, J. Robust clustering in generalized bounded confidence models. *J. Artif. Soc. Soc. Simul.* **19**, (2016).

4. Mäs, M. & Flache, A. Differentiation without distancing. Explaining bi-polarization of opinions without negative influence. *PloS One* **8**, e74516 (2013).

5. Friedkin, N. E., Proskurnikov, A. V. & Bullo, F. Group dynamics on multidimensional object threat appraisals. *Soc. Netw.* **65**, 157–167 (2021).

6. Kozitsin, I. V. Formal models of opinion formation and their application to real data: evidence from online social networks. *J. Math. Sociol.* (2020).

7. Kozitsin, I. V. Opinion dynamics of online social network users: a micro-level analysis. *J. Math. Sociol.* (2021).

8. Carpentras, D. & Quayle, M. *The sensitivity of the Deffuant model to measurement error*. (2021).

9. Takács, K., Flache, A. & Mäs, M. Discrepancy and Disliking Do Not Induce Negative Opinion Shifts. *PLOS ONE* **11**, e0157948 (2016).

10. Mastroeni, L., Vellucci, P. & Naldi, M. Agent-Based Models for Opinion Formation: A Bibliographic Survey. *IEEE Access* **7**, 58836–58848 (2019).

11. Kozitsin, I. V. *et al.* Modeling Political Preferences of Russian Users Exemplified by the Social Network Vkontakte. *Math. Models Comput. Simul.* **12**, 185–194 (2020).
